# The missing representatives of the hydrated sodium orthophosphate phases: Na_3_(PO_4_)(H_2_O)_7_ and Na_3_(PO_4_)(H_2_O)_6_

**DOI:** 10.1107/S2056989025009843

**Published:** 2025-11-11

**Authors:** Matthias Weil, Berthold Söger

**Affiliations:** a Institute for Chemical Technologies and Analytics, Division of Applied Solid State Chemistry, Getreidemarkt 9/E164-05-1, 1060 Vienna, Austria; b X-ray Centre, TU Wien, Getreidemarkt 9/E057-04, 1060 Vienna, Austria; University of Aberdeen, United Kingdom

**Keywords:** crystal structure, orthophosphate, hydrogen bonding, Na_3_PO_4_ hydrate

## Abstract

The crystal structures of the long-known compound Na_3_(PO_4_)(H_2_O)_6_, tris­odium orthophosphate hexa­hydrate, and the compound Na_3_(PO_4_)(H_2_O)_7_, tris­odium orthophosphate hepta­hydrate, the possible existence of which is discussed in the literature, were elucidated by single-crystal X-ray diffraction. In both crystal structures, all the water mol­ecules are bound to the sodium cations, but the different water content leads to different arrangements in terms of polyhedral linkage. In the case of the hepta­hydrate (space group *Pca*2_1_, *Z* = 4), this results in a layered structure made up from three sixfold coordinated Na^+^ cations with phosphate units in between. In the case of the hexa­hydrate (space group *P*1, *Z* = 4), a three-dimensional network is realised by one fivefold and five sixfold coordinated Na^+^ cations, in which the phosphate units are embedded in the voids. In both crystal structures, the water mol­ecules are involved in complex O—H⋯O hydrogen-bonding networks and form moderately strong hydrogen bonds on average, almost exclusively with the phosphate O atoms. It is noteworthy that some O atoms accept up to five such bonds.

## Abstract 2

Depending on their water content, the two phosphates Na_3_(PO_4_)(H_2_O)_*n*_ (*n* = 6, 7) exhibit different cross-linking of the polyhedra around the sodium cations, namely in the form of layers (*n* = 7) or a three-dimensional framework (*n* = 6).

## 1. Chemical context

In continuation of our structural studies on *M*_3_(*X*O_4_)(H_2_O)_*n*_ compounds with tetra­hedral anions (*M* = alkali metal; *X* = P, V), *viz*. K_3_(PO_4_)(D_2_O)_7_ (Weil & Stöger, 2020 ▸), K_3_(VO_4_)(H_2_O)_0.56_ and K_3_(VO_4_)(H_2_O)_4_ (Wolflehner & Weil, 2025 ▸), we became inter­ested in the Na_3_(PO_4_)(H_2_O)_*n*_ system. Trisodium phosphate (TSP) and its hydrated phases are industrially relevant chemicals used on a large scale in the saponification of fats, as cleaning agents, or as water softening agents (Schrödter *et al.*, 2008 ▸). They are also used as food additives for their properties as complexing agents, acidity regulators, melting salts, emulsifiers or firming agents. Together with other sodium phosphates they are listed under the European approval number E339 for food additives (European Commission, 2025 ▸). Although TSP and its hydrate phases are well investigated due to these areas of application, there are still contradictions in the literature regarding the existence and composition of some hydrate phases (Menzel & von Sahr, 1937 ▸; Ingerson & Morey, 1943 ▸; Quimby, 1947 ▸; Bell, 1949 ▸; Wendrow & Kobe, 1952 ▸, 1954 ▸). For example, a compound with composition ‘Na_3_(PO_4_)·12H_2_O’ is still offered in the chemical trade, even though it has long been known that a phase with this composition does not exist because it contains additional NaOH and must be reformulated as Na_3_PO_4_·(NaOH)_≃0.25_·12H_2_O (Tillmanns & Baur, 1970 ▸, 1971 ▸). Up to now, the existence of the hydrate phases Na_3_(PO_4_)(H_2_O)_8_, Na_3_(PO_4_)(H_2_O)_6_, and Na_3_(PO_4_)(H_2_O)_0.5_ has been unequivocally confirmed (Wendrow & Kobe, 1954 ▸), but only the crystal structures of the octa­hydrate (Larbot & Durand, 1983 ▸) and the hemihydrate (Averbuch-Pouchot & Durif, 1983 ▸) have been determined so far. In the older literature, the existence of Na_3_(PO_4_)(H_2_O)_7_ has been suggested by some authors (Menzel & von Sahr, 1937 ▸; Ingerson & Morey, 1943 ▸), but questioned by others (Quimby, 1947 ▸; Bell, 1949 ▸). In a more recent investigation of the thermal dehydration of ‘Na_3_(PO_4_)·12H_2_O’, the hepta­hydrate phase of TSP was reported to appear as an inter­mediate dehydration product as revealed by temperature-dependent Raman studies (Ghule *et al.*, 2001 ▸).

In this article, we report on the crystal structures of the long-known compound Na_3_(PO_4_)(H_2_O)_6_ and of the suspected Na_3_(PO_4_)(H_2_O)_7_, thereby confirming the existence of the hepta­hydrate phase of TSP.

## 2. Structural commentary

### 2.1. Na_3_(PO_4_)(H_2_O)_7_

**Figure 1:**
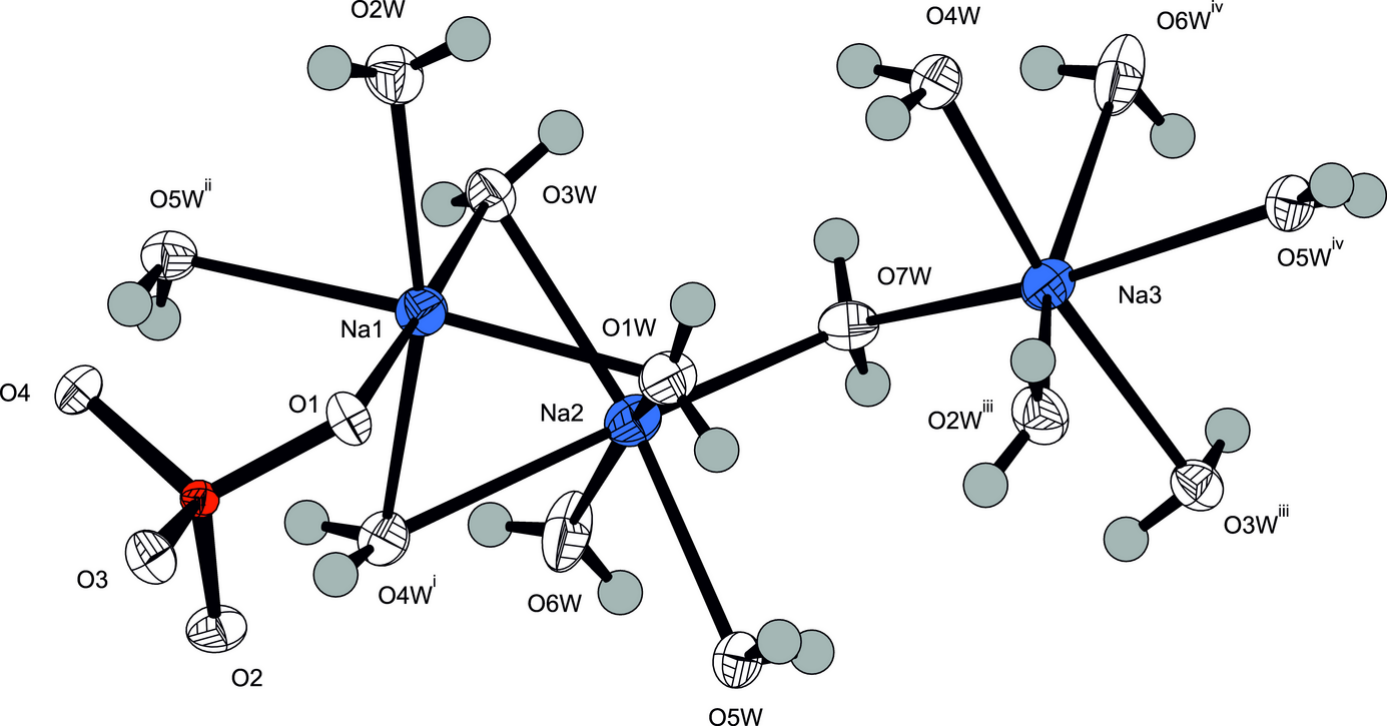
The asymmetric unit of Na_3_(PO_4_)(H_2_O)_7_ expanded to show the full coordination environments of the three Na^+^ cations. Displacement ellipsoid are given at the 90% probability level except for H atoms, which are shown with an arbitrary radius. Symmetry codes refer to Table 1 ▸.

**Figure 2:**
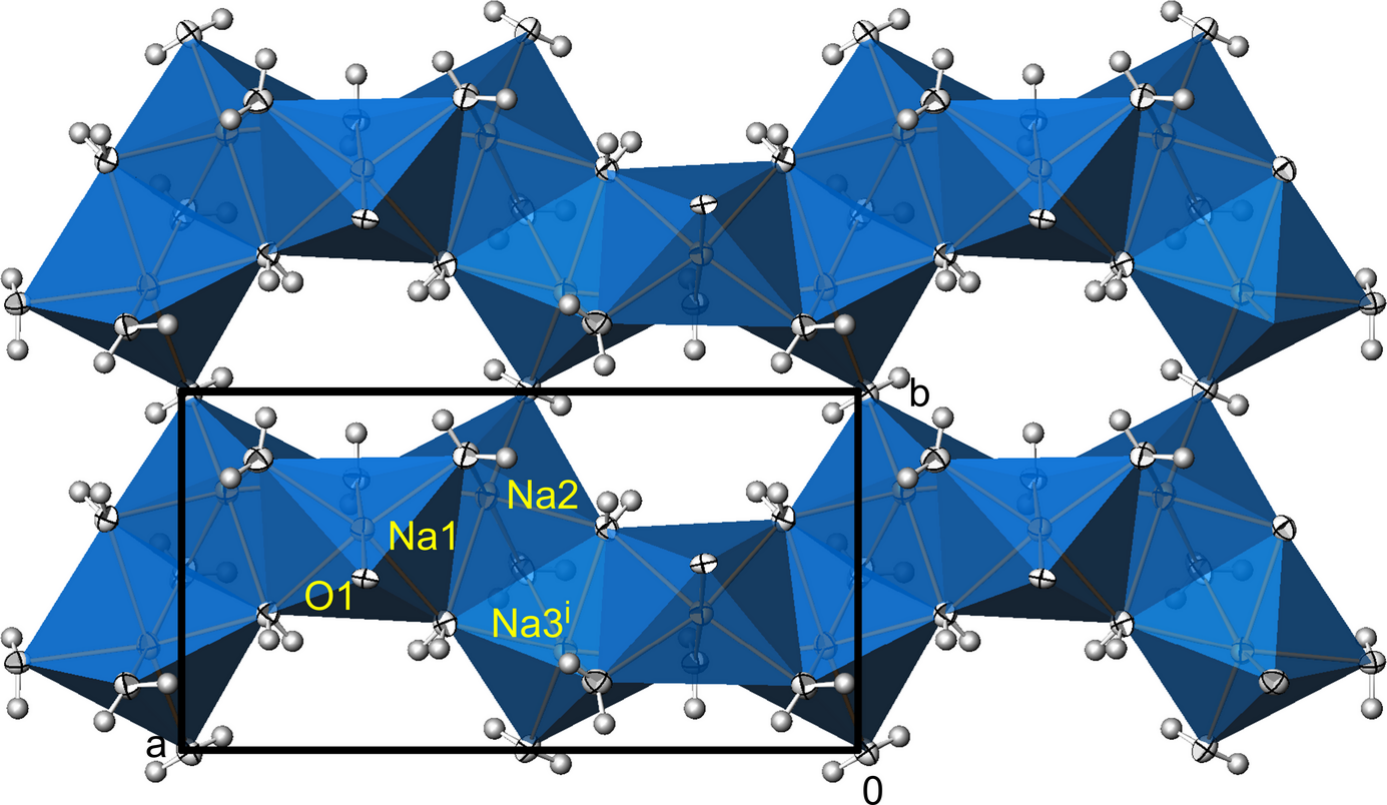
Na_3_(PO_4_)(H_2_O)_7_. Linkage of [NaO(H_2_O)_5_] (Na1) and [Na(H_2_O)_6_] (Na2, Na3) polyhedra into (001) layers in a view along [00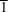]. Displacement ellipsoids are as in Fig. 1 ▸. [Symmetry code: (i) *x*, 1 − *y*, *z*.]

**Figure 3:**
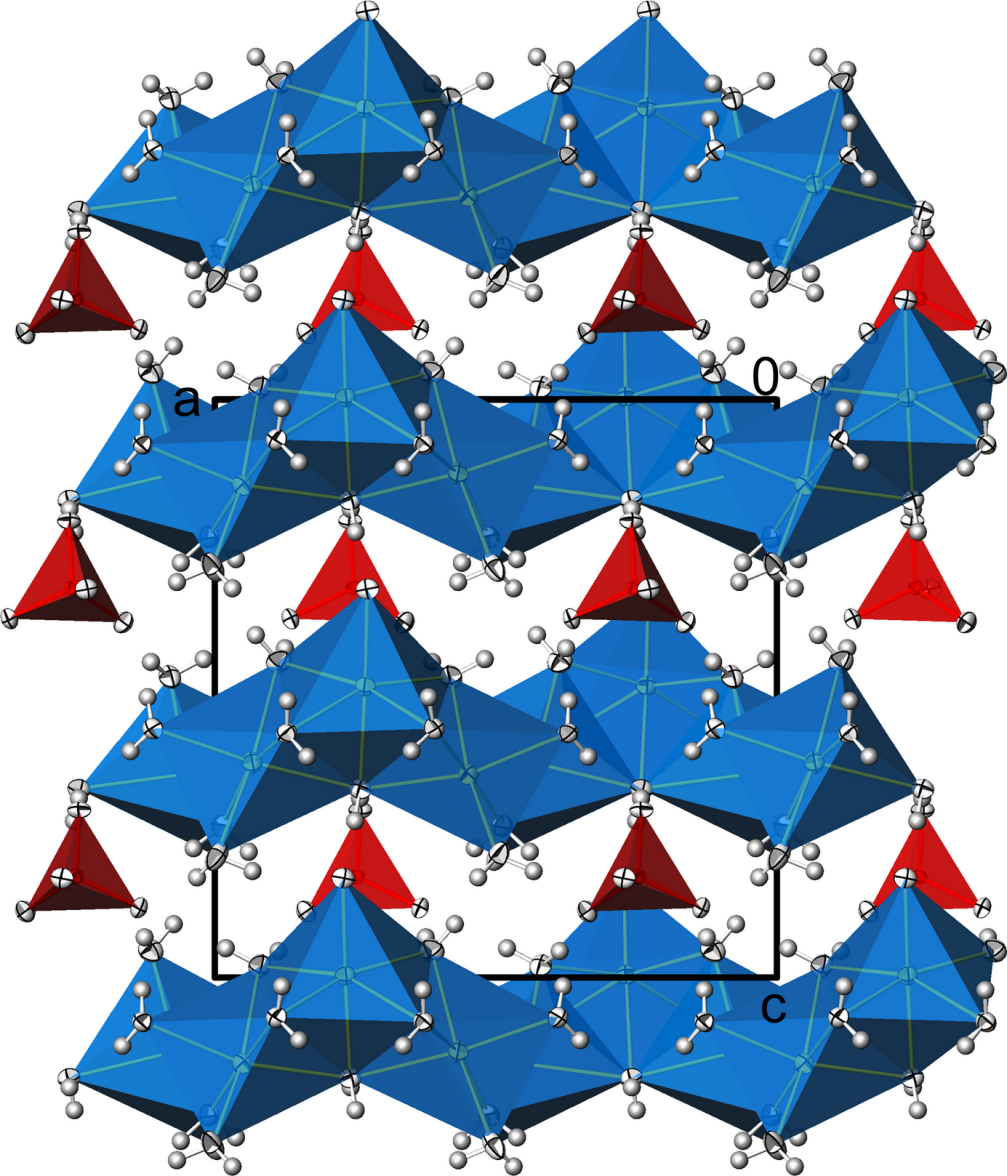
Na_3_(PO_4_)(H_2_O)_7_. View of the crystal structure along [0
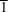
0], showing the [PO_4_]^3–^ tetra­hedra in between the cationic layers. Displacement ellipsoids are as in Fig. 1 ▸.

Na_3_(PO_4_)(H_2_O)_7_ crystallizes in the non-centrosymmetric ortho­rhom­bic space group *Pca*2_1_, and the absolute structure of the crystal chosen for data collection has been reliably determined [Flack parameter 0.00 (3)]. The asymmetric unit comprises one formula unit. The crystal structure consists of three Na^+^ cations sixfold surrounded by five water mol­ecules and one phosphate O atom for Na1, and by six water mol­ecules for both Na2 and Na3 (Fig. 1 ▸). By sharing edges and corners defined by water mol­ecules, these polyhedra are linked into a layer structure extending parallel to (001), Fig. 2 ▸. Thereby, three water mol­ecules (O*W*3, O*W*4, O*W*5) are bound to three Na^+^ cations, and four (O*W*1, O*W*2, O*W*6, O*W*7) to two Na^+^ cations each (Table 2). The isolated [PO_4_]^3–^ tetra­hedra are sandwiched between layers (Table 1 ▸ ▸, Fig. 3 ▸), with only one of the phosphate oxygen atoms (O1) directly bound to a sodium cation.

The description of the closest matching ideal coordination polyhedron for the three Na^+^ sites and qu­anti­fication of the distortion (*δ*) from it was performed with the *Polynator* program (Link & Niewa, 2023 ▸). In all cases, the idealized coordination polyhedron can be derived from a Bailar twist (dynamic) with moderate distortions (Table 2 ▸). The overall mean Na—O bond length in the three [NaO_6_] polyhedra amounts to 2.415 Å, in good agreement with the literature value of 2.441 (112) Å averaged from 920 individual polyhedra (Gagné & Hawthorne, 2016 ▸).

The P—O distances in the orthophosphate group lie in a narrow range (Table 2 ▸) with a mean of 1.546 Å, again in good agreement with the literature value of 1.537 (39) Å averaged from 3650 phosphate tetra­hedra (Gagné & Hawthorne, 2018 ▸). The slight angular distortions of the [PO_4_]^3–^ tetra­hedron is seen by the variation of the O—P—O angles ranging from 108.28 (3) to 111.12 (4)°.

**Figure 4:**
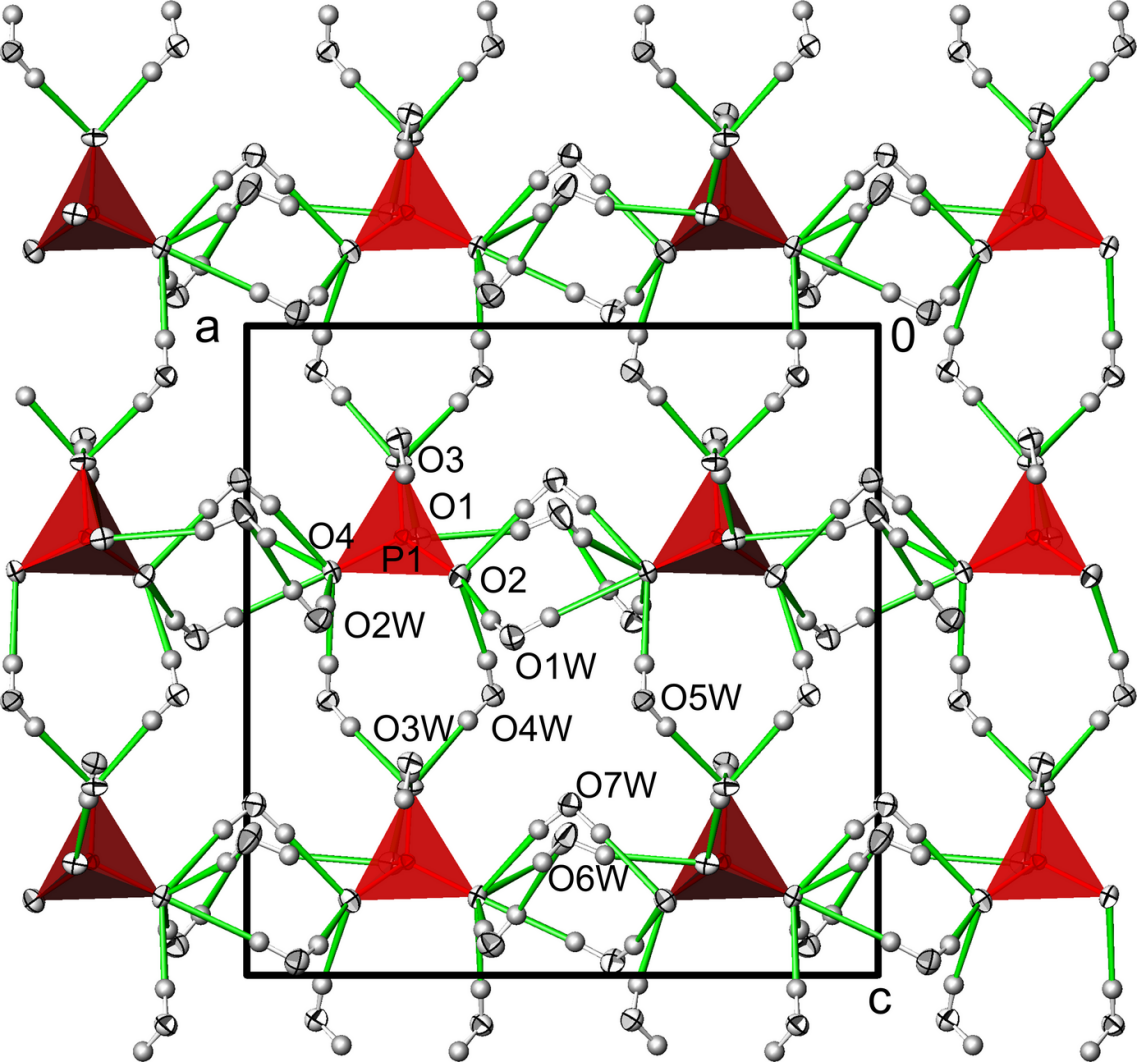
Na_3_(PO_4_)(H_2_O)_7_. Hydrogen-bonding network (green lines) between water mol­ecules and phosphate tetra­hedra. Displacement ellipsoids are as in Fig. 1 ▸.

The crystal structure of Na_3_(PO_4_)(H_2_O)_7_ is consolidated by an intricate network of O—H⋯O hydrogen bonds between water mol­ecules as donor groups and phosphate O atoms as acceptor atoms (Table 3 ▸, Fig. 4 ▸). All the water mol­ecules contribute to the hydrogen bonding with two approximately linear O—H⋯O links. The number of hydrogen bonds accepted differs for the O atoms of the phosphate group. Atom O1, which is the only one additionally bound to Na^+^, is the acceptor of two hydrogen bonds, O2 and O3 are each acceptors of three hydrogen bonds, while O4 is remarkably the acceptor of five hydrogen bonds. The *D*⋯*A* distances range from 2.5865 (9) to 3.2041 (11) Å, and on average can be classified as of medium strength (Jeffrey, 1997 ▸). It is noteworthy that only one hydrogen bond is formed with another water mol­ecule as the acceptor (O6*W*), namely with the longest observed *D*⋯*A* distance (Table 3 ▸).

### 2.2. Na_3_(PO_4_)(H_2_O)_6_

**Figure 5:**
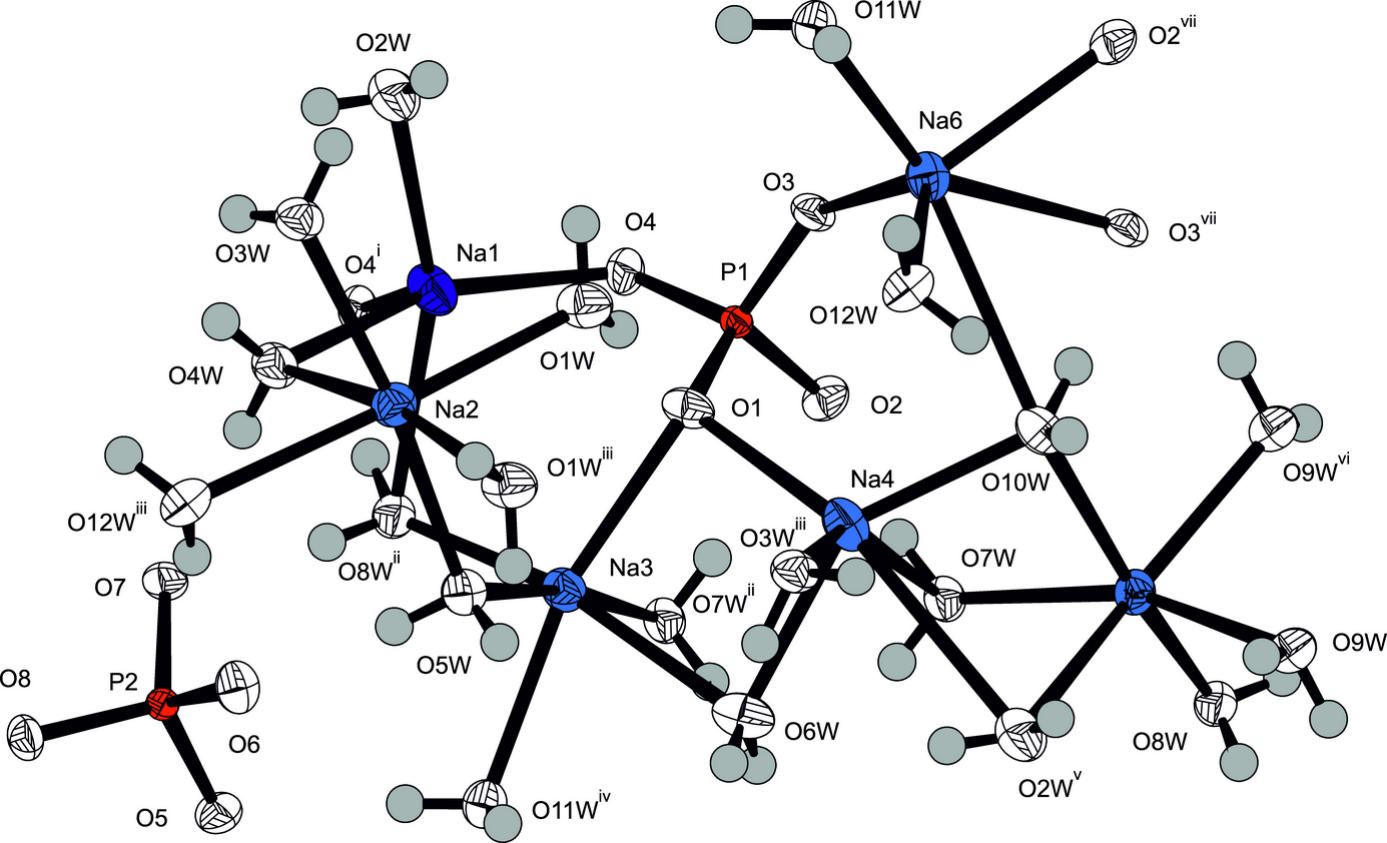
The asymmetric unit of Na_3_(PO_4_)(H_2_O)_6_ expanded to show the full coordination environments of the six Na^+^ cations. Displacement ellipsoid are as in Fig. 1 ▸. Symmetry codes refer to Table 4 ▸.

Na_3_(PO_4_)(H_2_O)_6_ crystallizes in the triclinic space group *P*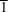 and comprises two formula units in the asymmetric unit. The crystal structure consists of one fivefold coordinated Na^+^ cation (Na1) and five sixfold coordinated Na^+^ cations (Na2–Na6) with different idealized coordination polyhedra (Fig. 5 ▸, Tables 2 ▸ and 4 ▸). The mean Na—O distance of the fivefold coordinated Na^+^ is 2.418 Å, in very good agreement with the literature value of 2.413 (108) Å (Gagné & Hawthorne, 2016 ▸). The total mean value of the Na—O distance of the five sixfold-coordinated Na^+^ cations is 2.455 Å, which is slightly longer than in the hepta­hydrate and corresponds almost perfectly with the value given in the literature (see above).

The lower water content compared to Na_3_(PO_4_)(H_2_O)_7_ can be seen in a lower number of coordinating water mol­ecules for the cations. Overall, only two of the six-coordinated cations (Na2, Na5) have all ligand atoms from water mol­ecules, two cations (Na3, Na4) have five water mol­ecules and one phosphate O atom in the coordination sphere, and one (Na6) has only three water mol­ecules and three phosphate O atoms; the five-coordinated Na1 also has three water mol­ecules as direct coordination partners. The individual polyhedra are in turn connected to each other by sharing corners and edges, with four of the water mol­ecules (O2*W*, O7*W*, O8*W*, O10*W*) bound to three cations simultaneously and the rest bound to two (Table 2 ▸).

**Figure 6:**
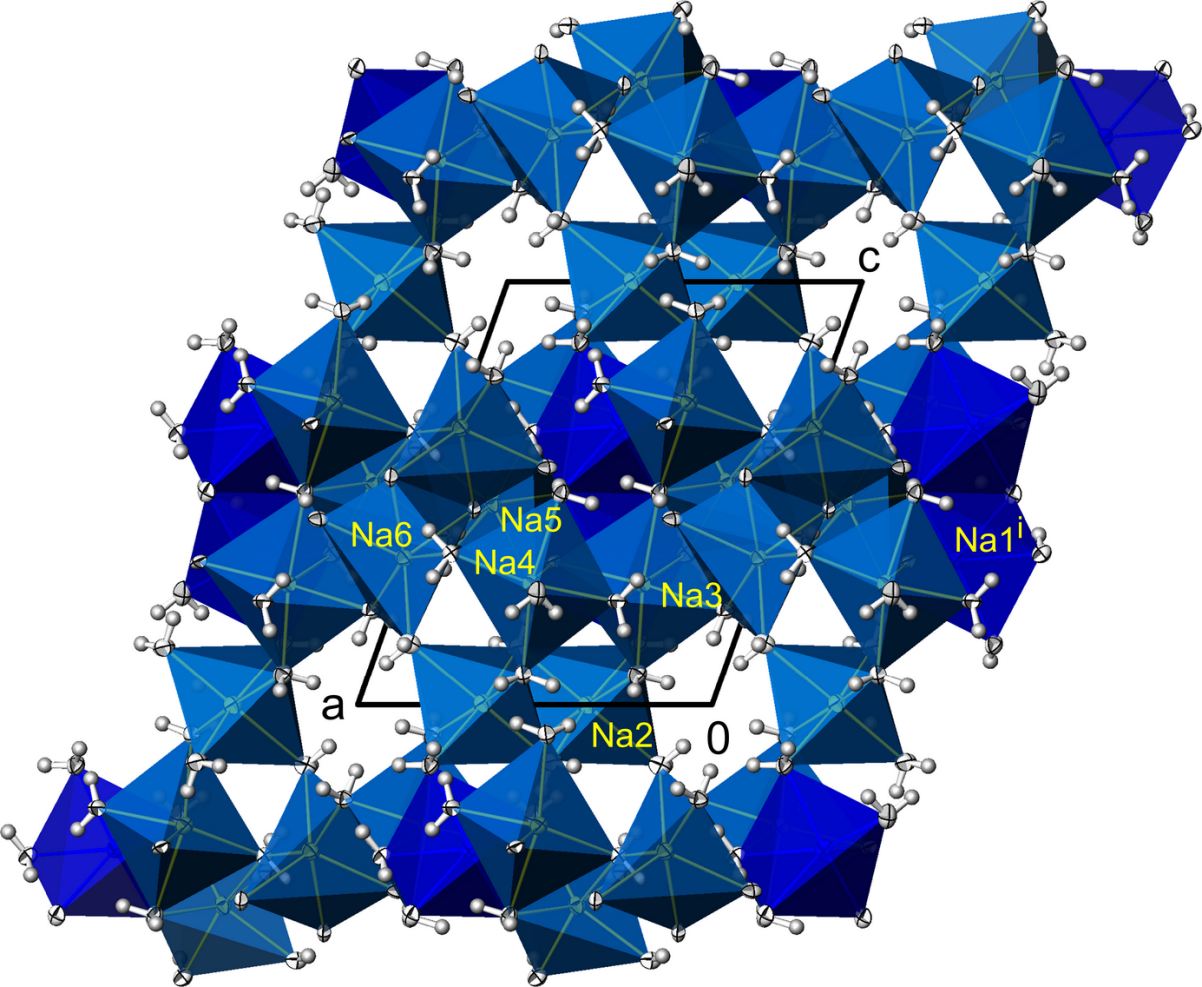
Na_3_(PO_4_)(H_2_O)_6_. Linkage of [NaO_2_(H_2_O)_3_] (Na1), [Na(H_2_O)_6_] (Na2, Na5), [NaO(H_2_O)_5_] (Na3, Na4), and [NaO_3_(H_2_O)_3_] (Na6) polyhedra into a framework structure in a view along [0
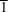
0]. Displacement ellipsoids are as in Fig. 1 ▸. [Symmetry code: (i) 1 − *x*, *y*, *z*.]

**Figure 7:**
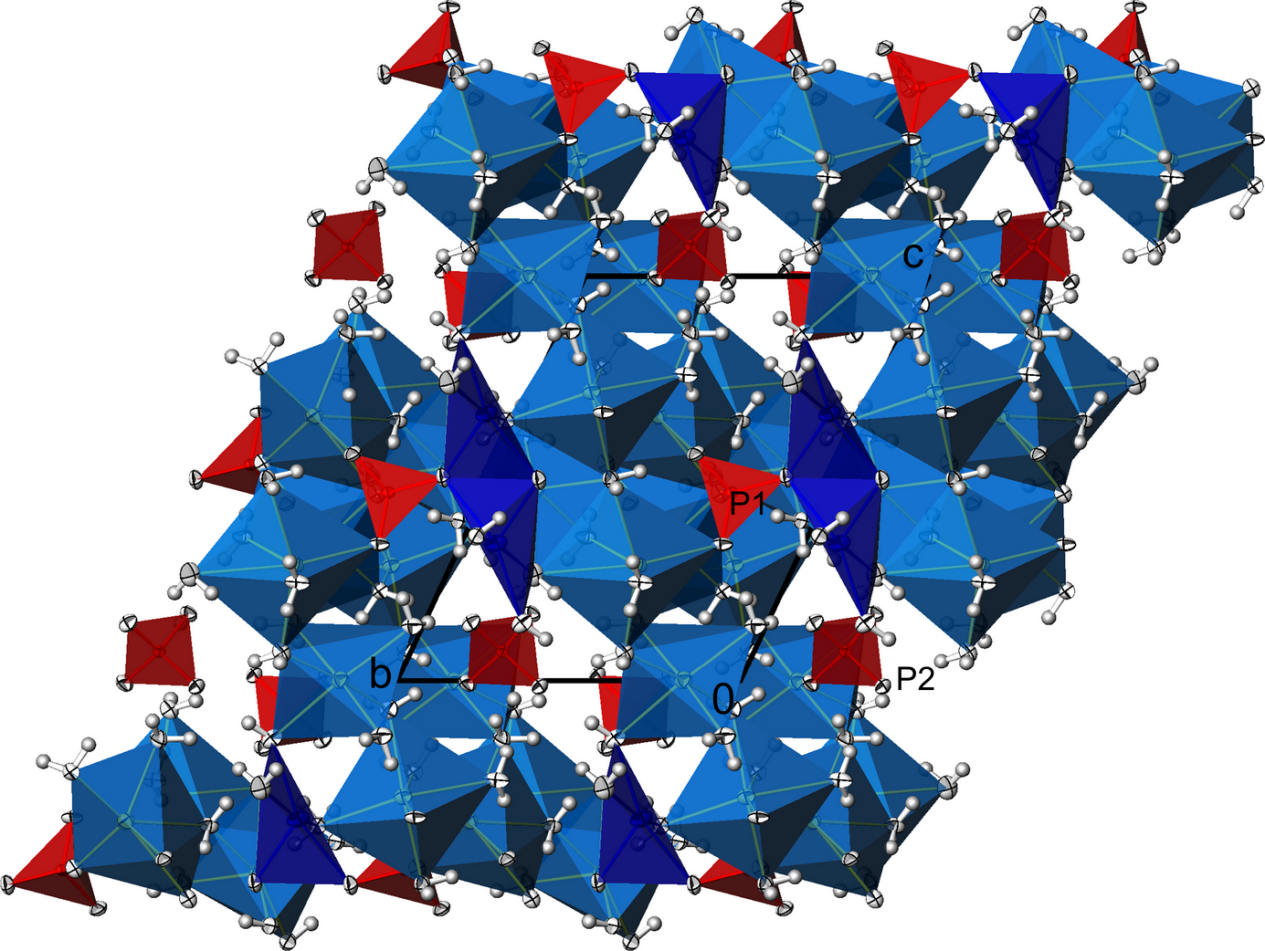
Na_3_(PO_4_)(H_2_O)_6_. View of the crystal structure along [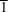
00] showing the [PO_4_]^3–^ tetra­hedra in the voids of the cationic framework. Displacement ellipsoids are as in Fig. 1 ▸.

Another difference to Na_3_(PO_4_)(H_2_O)_7_ concerns the resulting linkage of these polyhedra, which in this case is not in the form of layers but as a three-dimensional framework structure (Fig. 6 ▸). The [PO_4_]^3–^ tetra­hedra are isolated and located in the voids of this arrangement (Fig. 7 ▸). It is noteworthy here that the two unique [PO_4_]^3–^ tetra­hedra exhibit different properties. While all the O atoms of one tetra­hedron (P1) are also shared with Na^+^ cations, the O atoms of the other tetra­hedron (P2) belong exclusively to the P atom. However, these differences are not noticeable in the P—O bond lengths (Table 2 ▸). The range of P—O bond lengths is approximately the same in both phosphate tetra­hedra, and the respective mean values correspond to the literature value (see above). The range of O—P—O angles also does not differ significantly, ranging from 107.68 (3) to 110.03 (2)° for P1 and from 108.71 (3) to 110.23 (2)° for P2.

**Figure 8:**
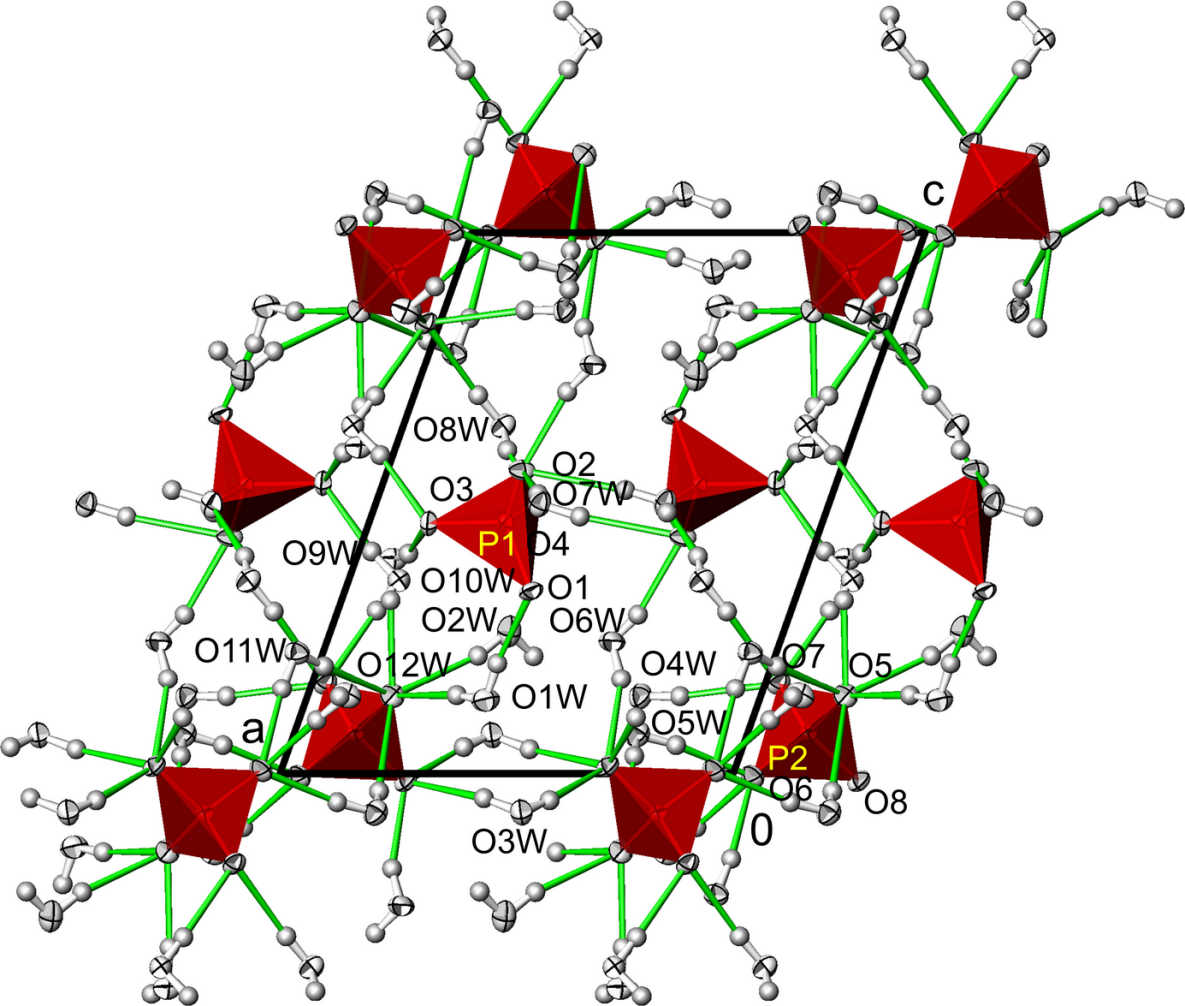
Na_3_(PO_4_)(H_2_O)_6_. Hydrogen-bonding network (green lines) between water mol­ecules and phosphate tetra­hedra. Displacement ellipsoids are as in Fig. 1 ▸.

A network of O—H⋯O hydrogen bonds consolidates the crystal structure of the hexa­hydrate and exhibit similar *D*⋯*A* distances and angles (Table 5 ▸, Fig. 8 ▸) as the hepta­hydrate. With only one water mol­ecule as an additional acceptor (O6*W*), the phosphate O atoms primarily assume this role in the hexa­hydrate as well. The differences between the two phosphate groups are clearly evident in the hydrogen-bonding network. The total number of accepted hydrogen bonds of seven for the P1 phosphate tetra­hedron (which also bonds to sodium ions) is significantly lower than for the ‘free’ P2 tetra­hedron with 15.

### 2.3. Bond valence sum calculation

Calculations of bond-valence sums (BVS; Brown, 2002 ▸) were performed with the program *ECoN21* (Ilinca, 2022 ▸) without contributions of H atoms. The BVS values of all atomic sites are listed in Table 2 ▸ and correspond to expectations for Na (1.0 valence unit, v. u.) and for P (5.0 v. u.). The BVS values obtained for the O atoms reflect their roles in the hydrogen-bonding networks. All water O atoms (O*W) have a value of less than 0.5 v.u., and all phosphate O atoms have a value significantly below the expected BVS value of 2.0 v.u., which is due to their role as acceptors of hydrogen bonds. There is a consistent trend for these phosphate O atoms showing that the BVS value decreases as the number of accepted hydrogen bonds increases (Tables 2 ▸, 3 ▸ and 5 ▸).

## 3. Database survey

A search of the Inorganic Crystal Structure Database (ICSD; data release 2025-1; Zagorac *et al.*, 2019 ▸) for Na_3_(*X*O_4_)(H_2_O)_*n*_ phases with tetra­hedral (*X*O_4_)^3–^ anions (*X* = P, As, V) revealed two entries for orthophosphates, Na_3_(PO_4_)(H_2_O)_8_ (Larbot & Durand, 1983 ▸) and Na_3_(PO_4_)(H_2_O)_0.5_ (Averbuch-Pouchot & Durif, 1983 ▸), no entry for orthoarsenates, and one entry for orthovanadates, Na_3_(VO_4_)(H_2_O)_3_ (Kato & Takayama-Muromachi, 1987 ▸).

Na_3_(PO_4_)(H_2_O)_8_ (space group *P*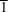) comprises two formula units in the asymmetric unit. From the six octa­hedrally surrounded Na^+^ cations, five exhibit solely water mol­ecules in the coordination sphere, and one four water mol­ecules and two O atoms from phosphate groups. Like in Na_3_(PO_4_)(H_2_O)_7_, the polyhedra around the Na^+^ cations are linked into a layered arrangement, with [PO_4_]^3–^ groups situated in between. Hydrogen atoms have not been determined for this structure, hence details on hydrogen-bonding inter­actions are limited to *D*⋯*A* distances.

Na_3_(PO_4_)(H_2_O)_0.5_ (space group *C*2/*c*) comprises one formula unit in the asymmetric unit. One of the three octa­hedrally surrounded Na^+^ cations has solely phosphate O atoms in the coordination sphere, while the other two have one water mol­ecule and five phosphate O atoms as ligands. Linking these polyhedra leads a framework structure. The water mol­ecule is situated on a twofold rotation axis and is the donor of two symmetry-related hydrogen bonds of medium strength.

Na_3_(VO_4_)(H_2_O)_3_ (space group *R*3) comprises one third of the formula unit in the asymmetric unit, with the V and one O atom situated on a threefold rotation axis. The [NaO_3_(H_2_O)_3_] octa­hedron shares its edges with neighbouring octa­hedra to form a framework structure. In this structure, too, two hydrogen bonds of medium strength are formed by the water mol­ecule.

## 4. Synthesis and crystallization

For the crystal growth of Na_3_(PO_4_)(H_2_O)_7_, a concentrated aqueous solution of Na_3_(PO_4_) was prepared in a polypropyl­ene beaker from commercially available Na_3_(PO_4_)(H_2_O)_6_ (Budenheim KG, Germany) and evaporated for one day at 361 K in a drying oven. Pieces were broken off from the compact product and crushed again by gentle pressing between two glass slides. Crystals were isolated under a polarizing microscope and tested on a single crystal diffractometer. Besides weakly diffracting multi-domain crystals of undetermined composition, high-quality crystals of Na_3_(PO_4_)(H_2_O)_7_ were obtained this way.

A suitable single crystals of Na_3_(PO_4_)(H_2_O)_6_ was taken directly from the storage container of a commercially available sample (Budenheim KG, Germany).

## 5. Refinement

Crystal data, data collection and structure refinement details are summarized in Table 6 ▸. To prevent possible water release, data collections were performed at 100 K. Experience has shown that measurements at low temperatures also enable better localization of hydrogen atom positions from difference-Fourier maps, which was the case for all hydrogen atoms in both hydrate phases. Their O—H bond lengths were refined with restraints using a value of 0.85 (1) Å. Quick measurements of crystals of both compounds at room temperature showed no notable differences from the low-temperature measurements.

## Supplementary Material

Crystal structure: contains datablock(s) Na3PO4H2O6, Na3PO4H2O7, global. DOI: 10.1107/S2056989025009843/hb8171sup1.cif

Structure factors: contains datablock(s) Na3PO4H2O6. DOI: 10.1107/S2056989025009843/hb8171Na3PO4H2O6sup2.hkl

Structure factors: contains datablock(s) Na3PO4H2O7. DOI: 10.1107/S2056989025009843/hb8171Na3PO4H2O7sup3.hkl

CCDC references: 2500666, 2500667

Additional supporting information:  crystallographic information; 3D view; checkCIF report

## Figures and Tables

**Table 1:** Selected bond lengths (Å) for Na_3_(PO_4_)(H_2_O)_7_

Na1—O1	2.3010 (8)	Na2—O3*W*	2.4146 (7)
Na1—O1*W*	2.3146 (7)	Na2—O5*W*	2.4279 (7)
Na1—O2*W*	2.3440 (7)	Na2—O4*W*^i^	2.6357 (7)
Na1—O3*W*	2.4413 (8)	Na3—O4*W*	2.3605 (7)
Na1—O4*W*^i^	2.4626 (7)	Na3—O7*W*	2.3606 (7)
Na1—O5*W*^ii^	2.5311 (7)	Na3—O2*W*^iii^	2.3656 (8)
Na2—O1*W*	2.2991 (7)	Na3—O3*W*^iii^	2.4421 (8)
Na2—O6*W*	2.3093 (8)	Na3—O5*W*^iv^	2.4439 (7)
Na2—O7*W*	2.3558 (7)	Na3—O6*W*^iv^	2.6575 (10)

Symmetry codes: (i) 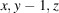; (ii) 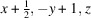; (iii) 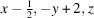; (iv) 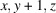.

**Table 2:** Coordination environments in Na_3_(PO_4_)(H_2_O)_7_ and Na_3_(PO_4_)(H_2_O)_6_, and results of BVS calculations (H atoms not taken into account)

Atom	Number of coordination partners	Polyhedron (idealized point group symmetry; deviation *δ* from it)	Range of *M*—O bond lengths (Å)	Average *M*—O bond length (Å)	Number of water mol­ecules in the first coordination sphere (Na—O < 3.0 Å); *number of accepted hydrogen bonds*	BVS (v.u.)
**Na_3_(PO_4_)(H_2_O)_7_**						
Na1	6	Bailar twist (dynamic) (32; 6.105)	2.3009 (8)–2.5307 (8)	2.399	5	1.19
Na2	6	Bailar twist (dynamic) (32; 7.566)	2.2989 (8)–2.6359 (8)	2.407	6	1.16
Na3	6	Bailar twist (dynamic) (32; 7.357)	2.3604 (8)–2.6575 (10)	2.438	6	1.08
P1	4	tetra­hedron ( 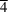 3*m*; 1.306)	1.5367 (6)–1.5655 (7)	1.546	–	4.93
O1	2				*2*	1.50
O2	1				*3*	1.26
O3	1				*3*	1.25
O4	1				*5*	1.17
O1*W*	2				–	0.47
O2*W*	2				–	0.43
O3*W*	3				–	0.53
O4*W*	3				–	0.48
O5*W*	3				–	0.49
O6*W*	2				*1*	0.36
O7*W*	2				–	0.43
						
**Na_3_(PO_4_)(H_2_O)_6_**						
Na1	5	Ψ-1 octa­hedron (4*mm*; 22.301)	2.3505 (6)–2.5944 (5)	2.418	3	0.98
Na2	6	twisted trigonal prism (32; 6.248)	2.3508 (5)–2.5269 (6)	2.428	6	1.08
Na3	6	twisted trigonal prism (32; 7.483)	2.3549 (5)–2.5365 (6)	2.433	5	1.07
Na4	6	trigonal anti­frustum (3*m*; 12.318)	2.3115 (5)–2.9741 (6)	2.485	5	1.08
Na5	6	twisted trigonal prism (32; 6.907)	2.3151 (5)–2.5838 (6)	2.395	6	1.18
Na6	6	isosceles wedge (*mm*2; 22.202)	2.3321 (5)–2.9641 (5)	2.533	3	0.96
P1	4	tetra­hedron ( 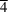 3*m*; 0.995)	1.5336 (5)–1.5525 (5)	1.544	–	4.92
P2	4	tetra­hedron ( 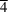 3*m*; 0.823)	1.5348 (5)–1.5509 (5)	1.545	–	4.95
O1	3				*1*	1.69
O2	2				*3*	1.41
O3	3				*2*	1.54
O4	3				*1*	1.70
O5	1				*5*	1.21
O6	1				*3*	1.26
O7	1				*4*	1.22
O8	1				*4*	1.21
O1*W*	2				–	0.41
O2*W*	3				–	0.36
O3*W*	2				–	0.42
O4*W*	2				–	0.34
O5*W*	2				–	0.43
O6*W*	2				*1*	0.36
O7*W*	3				–	0.52
O8*W*	3				–	0.49
O9*W*	2				–	0.46
O10*W*	3				–	0.48
O11*W*	2				–	0.36
O12*W*	2				–	0.35

**Table 3:** Hydrogen-bond geometry (Å, °) for Na_3_(PO_4_)(H_2_O)_7_

*D*—H⋯*A*	*D*—H	H⋯*A*	*D*⋯*A*	*D*—H⋯*A*
O1*W*—H1*WA*⋯O4^v^	0.84 (1)	2.09 (1)	2.9344 (9)	176 (2)
O1*W*—H1*WB*⋯O2^iv^	0.85 (1)	1.74 (1)	2.5865 (9)	172 (2)
O2*W*—H2*WA*⋯O6*W*^vi^	0.85 (1)	2.38 (1)	3.2042 (11)	163 (2)
O2*W*—H2*WB*⋯O4^iv^	0.84 (1)	1.93 (1)	2.7634 (9)	175 (2)
O3*W*—H3*WA*⋯O1^vii^	0.85 (1)	1.85 (1)	2.6867 (9)	168 (2)
O3*W*—H3*WB*⋯O3^viii^	0.87 (1)	1.88 (1)	2.7391 (9)	172 (2)
O4*W*—H4*WA*⋯O2^iv^	0.84 (1)	1.97 (1)	2.8058 (9)	173 (2)
O4*W*—H4*WB*⋯O3^viii^	0.86 (1)	1.84 (1)	2.6978 (9)	171 (2)
O5*W*—H5*WA*⋯O3^ix^	0.85 (1)	1.87 (1)	2.7114 (9)	173 (2)
O5*W*—H5*WB*⋯O4^v^	0.85 (1)	1.92 (1)	2.7544 (9)	169 (2)
O6*W*—H6*WA*⋯O1^ix^	0.85 (1)	1.99 (1)	2.8109 (9)	163 (2)
O6*W*—H6*WB*⋯O4^vii^	0.84 (1)	2.01 (1)	2.8323 (9)	169 (2)
O7*W*—H7*WA*⋯O4^viii^	0.85 (1)	2.02 (1)	2.8616 (9)	170 (2)
O7*W*—H7*WB*⋯O2^ix^	0.86 (1)	1.93 (1)	2.7894 (9)	175 (2)

Symmetry codes: (iv) 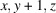; (v) 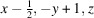; (vi) 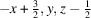; (vii) 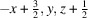; (viii) 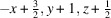; (ix) 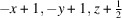.

**Table 4:** Selected bond lengths (Å) for Na_3_(PO_4_)(H_2_O)_6_

Na1—O4	2.3205 (6)	Na4—O1	2.3278 (5)
Na1—O4^i^	2.3722 (5)	Na4—O6*W*	2.3433 (5)
Na1—O4*W*	2.3907 (6)	Na4—O3*W*^iii^	2.3463 (5)
Na1—O2*W*	2.4107 (6)	Na4—O7*W*	2.6079 (6)
Na1—O8*W*^ii^	2.5944 (5)	Na4—O2*W*^v^	2.9741 (6)
Na2—O5*W*	2.3508 (5)	Na5—O7*W*	2.3151 (5)
Na2—O1*W*	2.3690 (6)	Na5—O9*W*	2.3155 (5)
Na2—O3*W*	2.3726 (5)	Na5—O9*W*^vi^	2.3577 (5)
Na2—O1*W*^iii^	2.4245 (6)	Na5—O8*W*	2.3971 (5)
Na2—O12*W*^iii^	2.5221 (6)	Na5—O10*W*	2.4006 (5)
Na2—O4*W*	2.5269 (6)	Na5—O2*W*^v^	2.5838 (6)
Na3—O5*W*	2.3549 (5)	Na6—O3	2.3321 (5)
Na3—O1	2.3707 (5)	Na6—O12*W*	2.3913 (5)
Na3—O8*W*^ii^	2.4357 (5)	Na6—O11*W*	2.4088 (5)
Na3—O11*W*^iv^	2.4372 (5)	Na6—O2^vii^	2.4443 (5)
Na3—O7*W*^ii^	2.4623 (5)	Na6—O3^vii^	2.6552 (6)
Na3—O6*W*	2.5365 (6)	Na6—O10*W*	2.9641 (5)
Na4—O10*W*	2.3115 (5)		

Symmetry codes: (i) 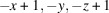; (ii) 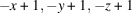; (iii) 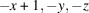; (iv) 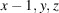; (v) 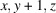; (vi) 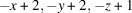; (vii) 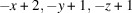.

**Table 5:** Hydrogen-bond geometry (Å, °) for Na_3_(PO_4_)(H_2_O)_6_

*D*—H⋯*A*	*D*—H	H⋯*A*	*D*⋯*A*	*D*—H⋯*A*
O1*W*—H1*WA*⋯O1	0.85 (1)	1.83 (1)	2.6704 (7)	171 (1)
O1*W*—H1*WB*⋯O5^viii^	0.86 (1)	2.09 (1)	2.9417 (6)	172 (1)
O2*W*—H2*WA*⋯O6*W*^ix^	0.82 (1)	2.47 (1)	3.1079 (8)	136 (1)
O2*W*—H2*WB*⋯O5^viii^	0.82 (1)	2.03 (1)	2.8343 (7)	167 (1)
O3*W*—H3*WA*⋯O8^x^	0.83 (1)	1.84 (1)	2.6651 (6)	175 (1)
O3*W*—H3*WB*⋯O8^viii^	0.85 (1)	2.03 (1)	2.8469 (6)	161 (1)
O4*W*—H4*WA*⋯O7	0.82 (1)	2.04 (1)	2.8552 (6)	173 (1)
O4*W*—H4*WB*⋯O8^x^	0.81 (1)	2.00 (1)	2.7987 (7)	170 (1)
O5*W*—H5*WA*⋯O6	0.84 (1)	1.80 (1)	2.6359 (7)	172 (1)
O5*W*—H5*WB*⋯O5^xi^	0.83 (1)	2.43 (1)	3.2184 (7)	159 (1)
O6*W*—H6*WA*⋯O8^xi^	0.84 (1)	1.95 (1)	2.7557 (6)	159 (1)
O6*W*—H6*WB*⋯O2^ii^	0.84 (1)	1.84 (1)	2.6508 (6)	160 (1)
O7*W*—H7*WA*⋯O2	0.86 (1)	1.77 (1)	2.6209 (6)	172 (1)
O7*W*—H7*WB*⋯O2^ii^	0.85 (1)	2.12 (1)	2.9658 (6)	174 (1)
O8*W*—H8*WA*⋯O7^ii^	0.83 (1)	1.95 (1)	2.7783 (7)	172 (1)
O8*W*—H8*WB*⋯O4^v^	0.84 (1)	1.81 (1)	2.6220 (6)	164 (1)
O9*W*—H9*WA*⋯O5^xii^	0.82 (1)	1.93 (1)	2.7531 (7)	174 (1)
O9*W*—H9*WB*⋯O3^v^	0.84 (1)	1.90 (1)	2.7235 (6)	167 (1)
O10*W*—H10*A*⋯O7^xii^	0.83 (1)	1.91 (1)	2.7345 (6)	175 (1)
O10*W*—H10*B*⋯O3^vii^	0.85 (1)	1.85 (1)	2.6902 (6)	171 (1)
O11*W*—H11*A*⋯O6^iii^	0.87 (1)	1.90 (1)	2.7666 (6)	173 (1)
O11*W*—H11*B*⋯O5^viii^	0.85 (1)	2.00 (1)	2.8460 (7)	176 (1)
O12*W*—H12*A*⋯O6^iii^	0.86 (1)	1.90 (1)	2.7438 (7)	168 (1)
O12*W*—H12*B*⋯O7^xii^	0.85 (1)	1.97 (1)	2.8165 (6)	171 (1)

Symmetry codes: (ii) 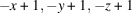; (iii) 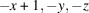; (v) 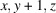; (vii) 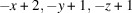; (viii) 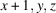; (ix) 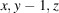; (x) 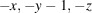; (xi) 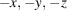; (xii) 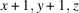.

**Table 6:** Experimental details

	Na_3_(PO_4_)(H_2_O)_7_	Na_3_(PO_4_)(H_2_O)_6_
Crystal data		
*M*_r_	290.05	272.04
Crystal system, space group	Orthorhombic, *Pca*2_1_	Triclinic, *P* 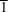
Temperature (K)	100	100
*a*, *b*, *c* (Å)	12.3169 (7), 6.5324 (3), 12.6602 (7)	9.5490 (4), 9.6353 (5), 12.1401 (6)
α, β, γ (°)	90, 90, 90	109.289 (4), 101.228 (4), 108.476 (4)
*V* (Å^3^)	1018.63 (9)	942.35 (8)
*Z*	4	4
Radiation type	Mo *K*α	Mo *K*α
μ (mm^−1^)	0.44	0.46
Crystal size (mm)	0.28 × 0.19 × 0.10	0.13 × 0.09 × 0.04

Data collection		
Diffractometer	Bruker APEXII CCD	Stoe STADIVARI
Absorption correction	Multi-scan (*SADABS*; Krause *et al.*, 2015 ▸)	Multi-scan (*LANA*; Koziskova *et al.*, 2016 ▸)
*T*_min_, *T*_max_	0.695, 0.748	0.911, 0.991
No. of measured, independent and observed [*I* > 2σ(*I*)] reflections	44325, 6462, 6108	42672, 9215, 8427
*R*_int_	0.032	0.018

Refinement		
*R*[*F*^2^ > 2σ(*F*^2^)], *wR*(*F*^2^), *S*	0.020, 0.046, 1.07	0.020, 0.056, 1.06
No. of reflections	6462	9215
No. of parameters	192	325
No. of restraints	15	24
H-atom treatment	All H-atom parameters refined	Only H-atom coordinates refined
Δρ_max_, Δρ_min_ (e Å^−3^)	0.32, −0.22	0.53, −0.32
Absolute structure	Flack *x* determined using 2767 quotients [(*I*^+^)−(*I*^−^)]/[(*I*^+^)+(*I*^−^)] (Parsons *et al.*, 2013 ▸)	–
Absolute structure parameter	0.00 (3)	–

Computer programs: *APEX3* and *SAINT* (Bruker, 2021 ▸), *X-AREA* (Stoe, 2024 ▸), *SHELXT* (Sheldrick, 2015*a* ▸), *SHELXL* (Sheldrick, 2015*b* ▸), *ATOMS* (Dowty, 2006 ▸) and *publCIF* (Westrip, 2010 ▸).

## supplementary crystallographic information

### Trisodium orthophosphate hexahydrate (Na3PO4H2O6) Crystal data

**Table:**

Na_3_(PO_4_)(H_2_O)_6_	*Z* = 4
*M**_r_* = 272.04	*F*(000) = 560
Triclinic, *P*1	*D*_x_ = 1.917 Mg m^−^^3^
*a* = 9.5490 (4) Å	Mo *K*α radiation, λ = 0.71073 Å
*b* = 9.6353 (5) Å	Cell parameters from 42515 reflections
*c* = 12.1401 (6) Å	θ = 1.9–36.7°
α = 109.289 (4)°	µ = 0.46 mm^−^^1^
β = 101.228 (4)°	*T* = 100 K
γ = 108.476 (4)°	Plate, colourless
*V* = 942.35 (8) Å^3^	0.13 × 0.09 × 0.04 mm

### Trisodium orthophosphate hexahydrate (Na3PO4H2O6) Data collection

**Table:**

Stoe STADIVARI diffractometer	9215 independent reflections
Radiation source: Axo_Mo	8427 reflections with *I* > 2σ(*I*)
Graded multilayer mirror monochromator	*R*_int_ = 0.018
Detector resolution: 13.33 pixels mm^-1^	θ_max_ = 36.9°, θ_min_ = 2.4°
rotation method, ω scans	*h* = −16→13
Absorption correction: multi-scan (*LANA*; Koziskova *et al.*, 2016)	*k* = −10→16
*T*_min_ = 0.911, *T*_max_ = 0.991	*l* = −20→20
42672 measured reflections	

### Trisodium orthophosphate hexahydrate (Na3PO4H2O6) Refinement

**Table:**

Refinement on *F*^2^	Primary atom site location: dual
Least-squares matrix: full	Hydrogen site location: difference Fourier map
*R*[*F*^2^ > 2σ(*F*^2^)] = 0.020	Only H-atom coordinates refined
*wR*(*F*^2^) = 0.056	*w* = 1/[σ^2^(*F*_o_^2^) + (0.0291*P*)^2^ + 0.1762*P*] where *P* = (*F*_o_^2^ + 2*F*_c_^2^)/3
*S* = 1.06	(Δ/σ)_max_ = 0.002
9215 reflections	Δρ_max_ = 0.53 e Å^−^^3^
325 parameters	Δρ_min_ = −0.32 e Å^−^^3^
24 restraints	

### Trisodium orthophosphate hexahydrate (Na3PO4H2O6) Special details

**Table:**

Geometry. All esds (except the esd in the dihedral angle between two l.s. planes) are estimated using the full covariance matrix. The cell esds are taken into account individually in the estimation of esds in distances, angles and torsion angles; correlations between esds in cell parameters are only used when they are defined by crystal symmetry. An approximate (isotropic) treatment of cell esds is used for estimating esds involving l.s. planes.

### Trisodium orthophosphate hexahydrate (Na3PO4H2O6) Fractional atomic coordinates and isotropic or equivalent isotropic displacement parameters (Å^2^)

**Table:**

	*x*	*y*	*z*	*U*_iso_*/*U*_eq_	
Na1	0.46040 (3)	−0.10173 (3)	0.34021 (2)	0.01131 (5)	
Na2	0.35186 (3)	−0.16635 (3)	−0.00188 (2)	0.00949 (5)	
Na3	0.31635 (3)	0.17465 (3)	0.28593 (2)	0.00889 (4)	
Na4	0.63254 (3)	0.45792 (3)	0.28635 (2)	0.01083 (5)	
Na5	0.81993 (3)	0.85412 (3)	0.47425 (2)	0.00871 (4)	
Na6	1.01416 (3)	0.39929 (3)	0.34657 (2)	0.00953 (5)	
P1	0.69885 (2)	0.28053 (2)	0.46564 (2)	0.00492 (3)	
P2	−0.13497 (2)	−0.26690 (2)	0.07362 (2)	0.00497 (3)	
O1	0.58849 (5)	0.23882 (5)	0.33732 (4)	0.00922 (7)	
O2	0.70340 (5)	0.43730 (5)	0.56120 (4)	0.00895 (7)	
O3	0.86741 (5)	0.31380 (5)	0.46279 (4)	0.00820 (7)	
O4	0.63891 (5)	0.14101 (5)	0.50280 (4)	0.00914 (7)	
O5	−0.18726 (5)	−0.13720 (5)	0.14385 (4)	0.00937 (7)	
O6	−0.04459 (5)	−0.20890 (5)	−0.00562 (4)	0.00942 (7)	
O7	−0.03024 (5)	−0.30105 (5)	0.16635 (4)	0.00814 (7)	
O8	−0.28289 (5)	−0.42346 (5)	−0.01384 (4)	0.00825 (7)	
O1W	0.60359 (5)	0.02983 (6)	0.13572 (4)	0.01135 (7)	
H1WA	0.6072 (14)	0.0949 (13)	0.2043 (10)	0.017*	
H1WB	0.6714 (13)	−0.0106 (14)	0.1449 (11)	0.017*	
O2W	0.60598 (6)	−0.25279 (6)	0.26337 (5)	0.01358 (8)	
H2WA	0.5326 (13)	−0.3403 (13)	0.2176 (11)	0.020*	
H2WB	0.6559 (14)	−0.2320 (15)	0.2186 (11)	0.020*	
O3W	0.43736 (5)	−0.37555 (5)	−0.07135 (4)	0.00970 (7)	
H3WA	0.3845 (13)	−0.4408 (13)	−0.0491 (10)	0.015*	
H3WB	0.5313 (12)	−0.3654 (13)	−0.0451 (10)	0.015*	
O4W	0.26544 (5)	−0.28566 (6)	0.14281 (4)	0.01040 (7)	
H4WA	0.1781 (12)	−0.2915 (14)	0.1430 (10)	0.016*	
H4WB	0.2588 (13)	−0.3752 (13)	0.1040 (10)	0.016*	
O5W	0.24137 (5)	0.01652 (5)	0.07208 (4)	0.01034 (7)	
H5WA	0.1484 (12)	−0.0496 (13)	0.0527 (10)	0.016*	
H5WB	0.2323 (13)	0.0709 (13)	0.0327 (10)	0.016*	
O6W	0.37090 (5)	0.42450 (6)	0.24429 (4)	0.01154 (8)	
H6WA	0.3214 (13)	0.4155 (14)	0.1753 (10)	0.017*	
H6WB	0.3349 (13)	0.4744 (14)	0.2944 (10)	0.017*	
O7W	0.63802 (5)	0.65073 (5)	0.49826 (4)	0.00930 (7)	
H7WA	0.6496 (13)	0.5753 (13)	0.5170 (10)	0.014*	
H7WB	0.5398 (12)	0.6220 (13)	0.4755 (10)	0.014*	
O8W	0.77912 (5)	1.03439 (5)	0.64277 (4)	0.00946 (7)	
H8WA	0.8590 (12)	1.1091 (13)	0.6996 (10)	0.014*	
H8WB	0.7518 (13)	1.0782 (13)	0.5982 (10)	0.014*	
O9W	0.91801 (5)	1.03959 (5)	0.39645 (4)	0.00958 (7)	
H9WA	0.8919 (13)	0.9917 (13)	0.3208 (9)	0.014*	
H9WB	0.8890 (13)	1.1149 (13)	0.4071 (10)	0.014*	
O10W	0.88846 (5)	0.64630 (6)	0.35754 (4)	0.00997 (7)	
H10A	0.9146 (13)	0.6684 (13)	0.3019 (10)	0.015*	
H10B	0.9719 (12)	0.6652 (13)	0.4111 (10)	0.015*	
O11W	1.05705 (5)	0.17297 (5)	0.22373 (4)	0.00941 (7)	
H11A	1.0618 (13)	0.1875 (13)	0.1572 (10)	0.014*	
H11B	0.9869 (12)	0.0787 (12)	0.1988 (10)	0.014*	
O12W	0.91400 (6)	0.38437 (6)	0.14452 (4)	0.01083 (7)	
H12A	0.9607 (13)	0.3419 (13)	0.0989 (10)	0.016*	
H12B	0.9286 (13)	0.4756 (12)	0.1426 (11)	0.016*	

### Trisodium orthophosphate hexahydrate (Na3PO4H2O6) Atomic displacement parameters (Å^2^)

**Table:**

	*U*^11^	*U*^22^	*U*^33^	*U*^12^	*U*^13^	*U*^23^
Na1	0.00846 (10)	0.01261 (11)	0.00943 (11)	0.00320 (9)	0.00247 (8)	0.00208 (9)
Na2	0.00993 (10)	0.00898 (10)	0.00958 (10)	0.00441 (8)	0.00319 (8)	0.00350 (8)
Na3	0.00822 (10)	0.00915 (10)	0.00819 (10)	0.00328 (8)	0.00196 (8)	0.00311 (8)
Na4	0.00839 (10)	0.01256 (11)	0.00898 (10)	0.00200 (9)	0.00190 (8)	0.00434 (9)
Na5	0.00863 (10)	0.00774 (10)	0.00979 (10)	0.00296 (8)	0.00392 (8)	0.00363 (8)
Na6	0.00947 (10)	0.00953 (10)	0.00881 (10)	0.00287 (8)	0.00378 (8)	0.00354 (8)
P1	0.00444 (5)	0.00515 (5)	0.00481 (5)	0.00190 (4)	0.00119 (4)	0.00196 (4)
P2	0.00499 (5)	0.00510 (5)	0.00470 (5)	0.00206 (4)	0.00134 (4)	0.00208 (4)
O1	0.00815 (16)	0.01242 (18)	0.00564 (15)	0.00500 (14)	0.00015 (13)	0.00263 (14)
O2	0.01142 (17)	0.00766 (16)	0.00709 (16)	0.00488 (14)	0.00300 (13)	0.00145 (13)
O3	0.00551 (14)	0.01020 (16)	0.01047 (17)	0.00352 (13)	0.00329 (13)	0.00557 (14)
O4	0.00861 (16)	0.00780 (16)	0.01155 (17)	0.00204 (13)	0.00348 (14)	0.00591 (14)
O5	0.00990 (16)	0.00825 (16)	0.00981 (17)	0.00520 (14)	0.00346 (14)	0.00214 (14)
O6	0.00998 (16)	0.01025 (17)	0.00833 (16)	0.00251 (14)	0.00420 (13)	0.00521 (14)
O7	0.00774 (15)	0.00951 (16)	0.00773 (16)	0.00397 (13)	0.00111 (13)	0.00471 (14)
O8	0.00684 (15)	0.00686 (15)	0.00772 (16)	0.00093 (13)	0.00056 (13)	0.00209 (13)
O1W	0.01197 (18)	0.01287 (18)	0.00871 (17)	0.00664 (15)	0.00221 (14)	0.00321 (15)
O2W	0.00987 (17)	0.0143 (2)	0.0168 (2)	0.00397 (15)	0.00499 (16)	0.00754 (17)
O3W	0.00861 (16)	0.01147 (18)	0.01137 (17)	0.00478 (14)	0.00439 (14)	0.00633 (15)
O4W	0.00843 (16)	0.00966 (17)	0.01095 (17)	0.00372 (14)	0.00291 (14)	0.00203 (14)
O5W	0.00952 (16)	0.00937 (17)	0.01091 (17)	0.00374 (14)	0.00255 (14)	0.00350 (14)
O6W	0.01125 (17)	0.0173 (2)	0.00671 (16)	0.00813 (16)	0.00269 (14)	0.00377 (15)
O7W	0.00787 (16)	0.00895 (16)	0.01092 (17)	0.00252 (13)	0.00286 (13)	0.00505 (14)
O8W	0.00934 (16)	0.00804 (16)	0.00966 (17)	0.00319 (14)	0.00179 (14)	0.00334 (14)
O9W	0.01174 (17)	0.00927 (17)	0.00822 (17)	0.00525 (14)	0.00296 (14)	0.00359 (14)
O10W	0.00814 (16)	0.01246 (18)	0.00823 (16)	0.00333 (14)	0.00267 (13)	0.00402 (14)
O11W	0.01033 (16)	0.00900 (17)	0.00891 (16)	0.00350 (14)	0.00338 (14)	0.00413 (14)
O12W	0.01409 (18)	0.00960 (17)	0.01098 (18)	0.00646 (15)	0.00509 (15)	0.00482 (14)

### Trisodium orthophosphate hexahydrate (Na3PO4H2O6) Geometric parameters (Å, º)

**Table:**

Na1—O4	2.3205 (6)	Na4—O2W^v^	2.9741 (6)
Na1—O4^i^	2.3722 (5)	Na5—O7W	2.3151 (5)
Na1—O4W	2.3907 (6)	Na5—O9W	2.3155 (5)
Na1—O2W	2.4107 (6)	Na5—O9W^vi^	2.3577 (5)
Na1—O8W^ii^	2.5944 (5)	Na5—O8W	2.3971 (5)
Na2—O5W	2.3508 (5)	Na5—O10W	2.4006 (5)
Na2—O1W	2.3690 (6)	Na5—O2W^v^	2.5838 (6)
Na2—O3W	2.3726 (5)	Na6—O3	2.3321 (5)
Na2—O1W^iii^	2.4245 (6)	Na6—O12W	2.3913 (5)
Na2—O12W^iii^	2.5221 (6)	Na6—O11W	2.4088 (5)
Na2—O4W	2.5269 (6)	Na6—O2^vii^	2.4443 (5)
Na3—O5W	2.3549 (5)	Na6—O3^vii^	2.6552 (6)
Na3—O1	2.3707 (5)	Na6—O10W	2.9641 (5)
Na3—O8W^ii^	2.4357 (5)	P1—O4	1.5336 (5)
Na3—O11W^iv^	2.4372 (5)	P1—O1	1.5419 (4)
Na3—O7W^ii^	2.4623 (5)	P1—O3	1.5482 (4)
Na3—O6W	2.5365 (6)	P1—O2	1.5525 (5)
Na4—O10W	2.3115 (5)	P2—O6	1.5348 (5)
Na4—O1	2.3278 (5)	P2—O7	1.5462 (4)
Na4—O6W	2.3433 (5)	P2—O5	1.5476 (4)
Na4—O3W^iii^	2.3463 (5)	P2—O8	1.5509 (5)
Na4—O7W	2.6079 (6)		
			
O1—P1—O2	108.70 (2)	P1—O2—Na6^vii^	100.64 (2)
O1—P1—O3	110.03 (2)	P1—O3—Na6	136.44 (3)
O3—P1—O2	107.68 (3)	P1—O3—Na6^vii^	92.37 (2)
O4—P1—O1	109.62 (3)	Na6—O3—Na6^vii^	95.452 (17)
O4—P1—O2	110.25 (3)	P1—O4—Na1^i^	136.46 (3)
O4—P1—O3	110.52 (2)	P1—O4—Na1	114.59 (3)
O5—P2—O8	108.71 (2)	Na1—O4—Na1^i^	97.030 (18)
O6—P2—O5	109.77 (3)	Na2—O1W—Na2^iii^	94.480 (19)
O6—P2—O7	109.83 (2)	Na2—O1W—H1WA	116.4 (8)
O6—P2—O8	108.30 (2)	Na2^iii^—O1W—H1WA	109.8 (8)
O7—P2—O5	110.23 (2)	Na2^iii^—O1W—H1WB	109.4 (8)
O7—P2—O8	109.96 (2)	Na2—O1W—H1WB	113.0 (8)
O4—Na1—O4^i^	82.970 (18)	H1WA—O1W—H1WB	112.2 (11)
O4—Na1—O2W	107.43 (2)	Na1—O2W—Na4^viii^	131.39 (2)
O4^i^—Na1—O2W	118.22 (2)	Na1—O2W—Na5^viii^	96.15 (2)
O4—Na1—O4W	159.42 (2)	Na1—O2W—H2WA	99.2 (8)
O4^i^—Na1—O4W	108.745 (19)	Na1—O2W—H2WB	121.8 (8)
O4—Na1—O8W^ii^	93.816 (18)	Na4^viii^—O2W—H2WA	63.3 (8)
O4^i^—Na1—O8W^ii^	63.542 (16)	Na4^viii^—O2W—H2WB	106.7 (8)
O4W—Na1—O2W	82.410 (19)	Na5^viii^—O2W—Na4^viii^	74.081 (16)
O4W—Na1—O8W^ii^	77.394 (17)	Na5^viii^—O2W—H2WA	134.0 (8)
O1W—Na2—O1W^iii^	85.520 (19)	Na5^viii^—O2W—H2WB	103.7 (9)
O1W—Na2—O3W	94.509 (18)	H2WA—O2W—H2WB	104.3 (12)
O1W^iii^—Na2—O4W	168.587 (19)	Na2—O3W—H3WA	100.2 (8)
O1W—Na2—O4W	98.343 (18)	Na2—O3W—H3WB	124.6 (7)
O1W^iii^—Na2—O12W^iii^	96.788 (18)	Na4^iii^—O3W—Na2	104.369 (19)
O1W—Na2—O12W^iii^	177.322 (19)	Na4^iii^—O3W—H3WA	115.0 (8)
O3W—Na2—O1W^iii^	103.980 (19)	Na4^iii^—O3W—H3WB	107.3 (8)
O3W—Na2—O12W^iii^	83.629 (18)	H3WA—O3W—H3WB	105.8 (11)
O5W—Na2—O1W^iii^	82.339 (18)	Na1—O4W—Na2	103.47 (2)
O5W—Na2—O1W	90.266 (19)	Na1—O4W—H4WA	111.4 (8)
O5W—Na2—O3W	172.34 (2)	Na1—O4W—H4WB	123.6 (8)
O5W—Na2—O4W	86.890 (18)	Na2—O4W—H4WA	111.5 (8)
O5W—Na2—O12W^iii^	91.388 (18)	Na2—O4W—H4WB	96.6 (8)
O1—Na3—O6W	87.697 (18)	H4WA—O4W—H4WB	108.8 (11)
O1—Na3—O7W^ii^	89.260 (18)	Na2—O5W—Na3	120.57 (2)
O1—Na3—O8W^ii^	103.275 (18)	Na2—O5W—H5WA	99.2 (8)
O1—Na3—O11W^iv^	164.843 (19)	Na2—O5W—H5WB	115.8 (8)
O5W—Na3—O1	98.056 (19)	Na3—O5W—H5WA	104.6 (8)
O5W—Na3—O6W	89.307 (18)	Na3—O5W—H5WB	110.4 (8)
O5W—Na3—O7W^ii^	171.710 (19)	H5WA—O5W—H5WB	103.3 (11)
O5W—Na3—O8W^ii^	100.594 (19)	Na3—O6W—H6WA	119.0 (8)
O5W—Na3—O11W^iv^	81.906 (18)	Na3—O6W—H6WB	99.5 (8)
O7W^ii^—Na3—O6W	87.087 (18)	Na4—O6W—Na3	87.121 (18)
O8W^ii^—Na3—O6W	163.889 (19)	Na4—O6W—H6WA	119.3 (8)
O8W^ii^—Na3—O7W^ii^	81.375 (17)	Na4—O6W—H6WB	125.4 (8)
O8W^ii^—Na3—O11W^iv^	91.578 (17)	H6WA—O6W—H6WB	104.4 (11)
O11W^iv^—Na3—O6W	77.146 (17)	Na3^ii^—O7W—Na4	169.88 (2)
O11W^iv^—Na3—O7W^ii^	90.011 (18)	Na3^ii^—O7W—H7WA	93.0 (7)
O1—Na4—O2W^v^	159.988 (18)	Na3^ii^—O7W—H7WB	94.6 (7)
O1—Na4—O3W^iii^	111.510 (19)	Na4—O7W—H7WA	80.0 (7)
O1—Na4—O6W	93.473 (19)	Na4—O7W—H7WB	94.1 (8)
O1—Na4—O7W	94.255 (18)	Na5—O7W—Na3^ii^	93.036 (19)
O3W^iii^—Na4—O2W^v^	78.629 (17)	Na5—O7W—Na4	85.941 (18)
O3W^iii^—Na4—O7W	147.855 (19)	Na5—O7W—H7WA	129.0 (7)
O6W—Na4—O2W^v^	70.391 (18)	Na5—O7W—H7WB	126.4 (7)
O6W—Na4—O3W^iii^	81.508 (18)	H7WA—O7W—H7WB	103.5 (10)
O6W—Na4—O7W	77.934 (18)	Na1^ii^—O8W—H8WA	108.7 (8)
O7W—Na4—O2W^v^	71.332 (16)	Na1^ii^—O8W—H8WB	63.4 (8)
O10W—Na4—O1	116.965 (19)	Na3^ii^—O8W—Na1^ii^	89.467 (17)
O10W—Na4—O2W^v^	75.406 (17)	Na3^ii^—O8W—H8WA	107.3 (8)
O10W—Na4—O3W^iii^	102.604 (19)	Na3^ii^—O8W—H8WB	144.2 (8)
O10W—Na4—O6W	144.02 (2)	Na5—O8W—Na1^ii^	131.86 (2)
O10W—Na4—O7W	80.946 (18)	Na5—O8W—Na3^ii^	91.707 (18)
O7W—Na5—O2W^v^	83.498 (18)	Na5—O8W—H8WA	116.7 (8)
O7W—Na5—O8W	85.300 (18)	Na5—O8W—H8WB	90.3 (8)
O7W—Na5—O9W	158.71 (2)	H8WA—O8W—H8WB	103.5 (11)
O7W—Na5—O9W^vi^	116.928 (19)	Na5—O9W—Na5^vi^	95.644 (19)
O7W—Na5—O10W	85.482 (18)	Na5^vi^—O9W—H9WA	121.6 (8)
O8W—Na5—O2W^v^	113.288 (19)	Na5—O9W—H9WA	108.6 (8)
O8W—Na5—O10W	161.013 (19)	Na5^vi^—O9W—H9WB	108.7 (8)
O9W^vi^—Na5—O2W^v^	153.12 (2)	Na5—O9W—H9WB	118.8 (8)
O9W—Na5—O2W^v^	76.393 (18)	H9WA—O9W—H9WB	104.4 (11)
O9W—Na5—O8W	96.191 (19)	Na4—O10W—Na5	91.035 (19)
O9W^vi^—Na5—O8W	87.022 (18)	Na4—O10W—Na6	92.274 (18)
O9W—Na5—O9W^vi^	84.355 (19)	Na4—O10W—H10A	112.3 (8)
O9W—Na5—O10W	98.413 (19)	Na4—O10W—H10B	133.7 (8)
O9W^vi^—Na5—O10W	82.422 (18)	Na5—O10W—Na6	149.66 (2)
O2^vii^—Na6—O3^vii^	58.627 (15)	Na5—O10W—H10A	108.0 (8)
O2^vii^—Na6—O10W	102.222 (17)	Na5—O10W—H10B	100.1 (8)
O3—Na6—O2^vii^	121.259 (19)	Na6—O10W—H10A	98.6 (8)
O3—Na6—O3^vii^	84.548 (17)	Na6—O10W—H10B	57.3 (7)
O3—Na6—O10W	90.449 (16)	H10A—O10W—H10B	106.6 (11)
O3^vii^—Na6—O10W	56.888 (14)	Na3^ix^—O11W—H11A	96.8 (7)
O3—Na6—O11W	107.102 (19)	Na3^ix^—O11W—H11B	110.2 (7)
O3—Na6—O12W	126.569 (19)	Na6—O11W—Na3^ix^	118.18 (2)
O11W—Na6—O2^vii^	90.615 (18)	Na6—O11W—H11A	103.7 (7)
O11W—Na6—O3^vii^	147.954 (18)	Na6—O11W—H11B	118.6 (8)
O11W—Na6—O10W	148.828 (18)	H11A—O11W—H11B	105.8 (10)
O12W—Na6—O2^vii^	111.333 (19)	Na2^iii^—O12W—H12A	92.3 (8)
O12W—Na6—O3^vii^	117.639 (18)	Na2^iii^—O12W—H12B	112.9 (8)
O12W—Na6—O10W	69.070 (16)	Na6—O12W—Na2^iii^	118.81 (2)
O12W—Na6—O11W	79.823 (17)	Na6—O12W—H12A	108.2 (8)
P1—O1—Na3	125.14 (3)	Na6—O12W—H12B	114.4 (8)
P1—O1—Na4	113.58 (3)	H12A—O12W—H12B	107.1 (11)
Na4—O1—Na3	91.501 (18)		

Symmetry codes: (i) −*x*+1, −*y*, −*z*+1; (ii) −*x*+1, −*y*+1, −*z*+1; (iii) −*x*+1, −*y*, −*z*; (iv) *x*−1, *y*, *z*; (v) *x*, *y*+1, *z*; (vi) −*x*+2, −*y*+2, −*z*+1; (vii) −*x*+2, −*y*+1, −*z*+1; (viii) *x*, *y*−1, *z*; (ix) *x*+1, *y*, *z*.

### Trisodium orthophosphate hexahydrate (Na3PO4H2O6) Hydrogen-bond geometry (Å, º)

**Table:**

*D*—H···*A*	*D*—H	H···*A*	*D*···*A*	*D*—H···*A*
O1*W*—H1*WA*···O1	0.85 (1)	1.83 (1)	2.6704 (7)	171 (1)
O1*W*—H1*WB*···O5^ix^	0.86 (1)	2.09 (1)	2.9417 (6)	172 (1)
O2*W*—H2*WA*···O6*W*^viii^	0.82 (1)	2.47 (1)	3.1079 (8)	136 (1)
O2*W*—H2*WB*···O5^ix^	0.82 (1)	2.03 (1)	2.8343 (7)	167 (1)
O3*W*—H3*WA*···O8^x^	0.83 (1)	1.84 (1)	2.6651 (6)	175 (1)
O3*W*—H3*WB*···O8^ix^	0.85 (1)	2.03 (1)	2.8469 (6)	161 (1)
O4*W*—H4*WA*···O7	0.82 (1)	2.04 (1)	2.8552 (6)	173 (1)
O4*W*—H4*WB*···O8^x^	0.81 (1)	2.00 (1)	2.7987 (7)	170 (1)
O5*W*—H5*WA*···O6	0.84 (1)	1.80 (1)	2.6359 (7)	172 (1)
O5*W*—H5*WB*···O5^xi^	0.83 (1)	2.43 (1)	3.2184 (7)	159 (1)
O6*W*—H6*WA*···O8^xi^	0.84 (1)	1.95 (1)	2.7557 (6)	159 (1)
O6*W*—H6*WB*···O2^ii^	0.84 (1)	1.84 (1)	2.6508 (6)	160 (1)
O7*W*—H7*WA*···O2	0.86 (1)	1.77 (1)	2.6209 (6)	172 (1)
O7*W*—H7*WB*···O2^ii^	0.85 (1)	2.12 (1)	2.9658 (6)	174 (1)
O8*W*—H8*WA*···O7^ii^	0.83 (1)	1.95 (1)	2.7783 (7)	172 (1)
O8*W*—H8*WB*···O4^v^	0.84 (1)	1.81 (1)	2.6220 (6)	164 (1)
O9*W*—H9*WA*···O5^xii^	0.82 (1)	1.93 (1)	2.7531 (7)	174 (1)
O9*W*—H9*WB*···O3^v^	0.84 (1)	1.90 (1)	2.7235 (6)	167 (1)
O10*W*—H10*A*···O7^xii^	0.83 (1)	1.91 (1)	2.7345 (6)	175 (1)
O10*W*—H10*B*···O3^vii^	0.85 (1)	1.85 (1)	2.6902 (6)	171 (1)
O11*W*—H11*A*···O6^iii^	0.87 (1)	1.90 (1)	2.7666 (6)	173 (1)
O11*W*—H11*B*···O5^ix^	0.85 (1)	2.00 (1)	2.8460 (7)	176 (1)
O12*W*—H12*A*···O6^iii^	0.86 (1)	1.90 (1)	2.7438 (7)	168 (1)
O12*W*—H12*B*···O7^xii^	0.85 (1)	1.97 (1)	2.8165 (6)	171 (1)

Symmetry codes: (ii) −*x*+1, −*y*+1, −*z*+1; (iii) −*x*+1, −*y*, −*z*; (v) *x*, *y*+1, *z*; (vii) −*x*+2, −*y*+1, −*z*+1; (viii) *x*, *y*−1, *z*; (ix) *x*+1, *y*, *z*; (x) −*x*, −*y*−1, −*z*; (xi) −*x*, −*y*, −*z*; (xii) *x*+1, *y*+1, *z*.

### Trisodium orthophosphate heptahydrate (Na3PO4H2O7) Crystal data

**Table:**

Na_3_(PO_4_)(H_2_O)_7_	*D*_x_ = 1.891 Mg m^−^^3^
*M**_r_* = 290.05	Mo *K*α radiation, λ = 0.71073 Å
Orthorhombic, *P**c**a*2_1_	Cell parameters from 9336 reflections
*a* = 12.3169 (7) Å	θ = 3.1–40.1°
*b* = 6.5324 (3) Å	µ = 0.44 mm^−^^1^
*c* = 12.6602 (7) Å	*T* = 100 K
*V* = 1018.63 (9) Å^3^	Fragment, colourless
*Z* = 4	0.28 × 0.19 × 0.10 mm
*F*(000) = 600	

### Trisodium orthophosphate heptahydrate (Na3PO4H2O7) Data collection

**Table:**

Bruker APEXII CCD diffractometer	6108 reflections with *I* > 2σ(*I*)
ω– and φ–scans	*R*_int_ = 0.032
Absorption correction: multi-scan (*SADABS*; Krause *et al.*, 2015)	θ_max_ = 40.5°, θ_min_ = 3.1°
*T*_min_ = 0.695, *T*_max_ = 0.748	*h* = −22→22
44325 measured reflections	*k* = −11→11
6462 independent reflections	*l* = −23→23

### Trisodium orthophosphate heptahydrate (Na3PO4H2O7) Refinement

**Table:**

Refinement on *F*^2^	Hydrogen site location: difference Fourier map
Least-squares matrix: full	All H-atom parameters refined
*R*[*F*^2^ > 2σ(*F*^2^)] = 0.020	*w* = 1/[σ^2^(*F*_o_^2^) + (0.0225*P*)^2^ + 0.0222*P*] where *P* = (*F*_o_^2^ + 2*F*_c_^2^)/3
*wR*(*F*^2^) = 0.046	(Δ/σ)_max_ = 0.002
*S* = 1.07	Δρ_max_ = 0.32 e Å^−^^3^
6462 reflections	Δρ_min_ = −0.22 e Å^−^^3^
192 parameters	Absolute structure: Flack *x* determined using 2767 quotients [(*I*^+^)-(*I*^-^)]/[(*I*^+^)+(*I*^-^)] (Parsons *et al.*, 2013).
15 restraints	Absolute structure parameter: 0.00 (3)

### Trisodium orthophosphate heptahydrate (Na3PO4H2O7) Special details

**Table:**

Geometry. All esds (except the esd in the dihedral angle between two l.s. planes) are estimated using the full covariance matrix. The cell esds are taken into account individually in the estimation of esds in distances, angles and torsion angles; correlations between esds in cell parameters are only used when they are defined by crystal symmetry. An approximate (isotropic) treatment of cell esds is used for estimating esds involving l.s. planes.

### Trisodium orthophosphate heptahydrate (Na3PO4H2O7) Fractional atomic coordinates and isotropic or equivalent isotropic displacement parameters (Å^2^)

**Table:**

	*x*	*y*	*z*	*U*_iso_*/*U*_eq_	
Na1	0.73222 (3)	0.61661 (6)	0.49574 (3)	0.00846 (6)	
Na2	0.54849 (3)	0.70865 (6)	0.64923 (3)	0.00916 (6)	
Na3	0.43297 (3)	1.27728 (6)	0.62867 (3)	0.00944 (6)	
P1	0.75326 (2)	0.24872 (3)	0.32554 (2)	0.00477 (3)	
O1	0.72833 (5)	0.47911 (9)	0.32845 (5)	0.00800 (9)	
O2	0.66276 (5)	0.13564 (9)	0.38566 (5)	0.00924 (9)	
O3	0.75902 (5)	0.16945 (10)	0.21082 (5)	0.00796 (9)	
O4	0.86543 (5)	0.20695 (9)	0.37948 (5)	0.00788 (9)	
O1W	0.57966 (5)	0.82117 (10)	0.47986 (5)	0.01029 (10)	
H1WA	0.5198 (11)	0.815 (3)	0.4480 (14)	0.028 (4)*	
H1WB	0.6126 (15)	0.921 (2)	0.4517 (17)	0.041 (6)*	
O2W	0.88619 (5)	0.81072 (10)	0.45322 (5)	0.01096 (10)	
H2WA	0.922 (2)	0.755 (3)	0.4035 (17)	0.057 (7)*	
H2WB	0.8770 (15)	0.928 (2)	0.4281 (15)	0.035 (5)*	
O3W	0.74117 (5)	0.75613 (10)	0.67442 (6)	0.00859 (10)	
H3WA	0.7496 (13)	0.683 (3)	0.7294 (12)	0.019 (4)*	
H3WB	0.7415 (12)	0.8842 (19)	0.6921 (16)	0.018 (4)*	
O4W	0.60995 (5)	1.35459 (9)	0.56927 (5)	0.00912 (9)	
H4WA	0.6198 (14)	1.287 (2)	0.5141 (11)	0.021 (4)*	
H4WB	0.6484 (15)	1.283 (2)	0.6127 (14)	0.027 (5)*	
O5W	0.37190 (5)	0.61919 (9)	0.57759 (5)	0.00872 (9)	
H5WA	0.3356 (15)	0.691 (3)	0.6207 (13)	0.025 (4)*	
H5WB	0.3699 (16)	0.688 (3)	0.5207 (12)	0.033 (5)*	
O6W	0.49639 (5)	0.50744 (10)	0.79038 (7)	0.01433 (12)	
H6WA	0.4324 (11)	0.504 (3)	0.8144 (15)	0.033 (5)*	
H6WB	0.5315 (13)	0.419 (2)	0.8240 (15)	0.030 (5)*	
O7W	0.48839 (5)	1.00429 (9)	0.73831 (5)	0.00970 (10)	
H7WA	0.5377 (13)	1.056 (3)	0.7769 (14)	0.034 (5)*	
H7WB	0.4388 (13)	0.965 (3)	0.7820 (13)	0.027 (4)*	

### Trisodium orthophosphate heptahydrate (Na3PO4H2O7) Atomic displacement parameters (Å^2^)

**Table:**

	*U*^11^	*U*^22^	*U*^33^	*U*^12^	*U*^13^	*U*^23^
Na1	0.00824 (13)	0.00899 (14)	0.00816 (14)	−0.00015 (11)	0.00008 (11)	−0.00087 (12)
Na2	0.00823 (13)	0.01088 (14)	0.00835 (15)	−0.00081 (11)	0.00028 (11)	0.00097 (12)
Na3	0.00783 (13)	0.01056 (14)	0.00992 (15)	−0.00032 (11)	−0.00038 (11)	0.00150 (12)
P1	0.00497 (6)	0.00469 (6)	0.00464 (6)	−0.00022 (5)	0.00001 (6)	0.00026 (6)
O1	0.0102 (2)	0.00554 (19)	0.0083 (2)	0.00096 (15)	0.0004 (2)	0.00007 (19)
O2	0.0082 (2)	0.0103 (2)	0.0092 (2)	−0.00266 (18)	0.00143 (19)	0.0019 (2)
O3	0.0108 (2)	0.0076 (2)	0.0055 (2)	0.00040 (17)	−0.00007 (17)	−0.00092 (18)
O4	0.0058 (2)	0.0095 (2)	0.0083 (2)	0.00073 (17)	−0.00134 (17)	0.00040 (19)
O1W	0.0096 (2)	0.0104 (2)	0.0110 (3)	−0.00099 (18)	−0.0012 (2)	0.0037 (2)
O2W	0.0125 (2)	0.0097 (2)	0.0107 (3)	−0.0005 (2)	0.0010 (2)	0.0026 (2)
O3W	0.0102 (2)	0.0075 (2)	0.0081 (2)	0.00052 (17)	−0.00060 (18)	−0.00046 (18)
O4W	0.0087 (2)	0.0099 (2)	0.0088 (2)	0.00065 (17)	−0.00025 (19)	0.0004 (2)
O5W	0.0086 (2)	0.0095 (2)	0.0080 (2)	0.00113 (17)	0.0008 (2)	0.0004 (2)
O6W	0.0109 (2)	0.0158 (3)	0.0163 (3)	0.0056 (2)	0.0050 (2)	0.0084 (2)
O7W	0.0093 (2)	0.0105 (2)	0.0093 (3)	−0.0020 (2)	0.00029 (19)	0.00024 (18)

### Trisodium orthophosphate heptahydrate (Na3PO4H2O7) Geometric parameters (Å, º)

**Table:**

Na1—O1	2.3010 (8)	Na2—O4W^i^	2.6357 (7)
Na1—O1W	2.3146 (7)	Na3—O4W	2.3605 (7)
Na1—O2W	2.3440 (7)	Na3—O7W	2.3606 (7)
Na1—O3W	2.4413 (8)	Na3—O2W^iii^	2.3656 (8)
Na1—O4W^i^	2.4626 (7)	Na3—O3W^iii^	2.4421 (8)
Na1—O5W^ii^	2.5311 (7)	Na3—O5W^iv^	2.4439 (7)
Na2—O1W	2.2991 (7)	Na3—O6W^iv^	2.6575 (10)
Na2—O6W	2.3093 (8)	P1—O1	1.5365 (6)
Na2—O7W	2.3558 (7)	P1—O2	1.5386 (6)
Na2—O3W	2.4146 (7)	P1—O3	1.5435 (7)
Na2—O5W	2.4279 (7)	P1—O4	1.5652 (6)
			
O1—Na1—O1W	97.38 (3)	Na2—O1W—H1WA	106.5 (13)
O1—Na1—O2W	90.95 (3)	Na1—O1W—H1WA	136.3 (14)
O1W—Na1—O2W	108.94 (3)	Na2—O1W—H1WB	135.9 (15)
O1—Na1—O3W	178.26 (3)	Na1—O1W—H1WB	95.4 (13)
O1W—Na1—O3W	84.36 (3)	H1WA—O1W—H1WB	104.9 (19)
O2W—Na1—O3W	88.53 (3)	Na1—O2W—Na3^v^	81.37 (2)
O1—Na1—O4W^i^	93.66 (2)	Na1—O2W—H2WA	110.7 (17)
O1W—Na1—O4W^i^	86.42 (2)	Na3^v^—O2W—H2WA	117.8 (19)
O2W—Na1—O4W^i^	163.26 (3)	Na1—O2W—H2WB	118.3 (13)
O3W—Na1—O4W^i^	86.38 (2)	Na3^v^—O2W—H2WB	127.7 (14)
O1—Na1—O5W^ii^	98.83 (2)	H2WA—O2W—H2WB	101 (2)
O1W—Na1—O5W^ii^	159.85 (3)	Na2—O3W—Na1	77.60 (2)
O2W—Na1—O5W^ii^	82.72 (2)	Na2—O3W—Na3^v^	155.23 (4)
O3W—Na1—O5W^ii^	79.46 (2)	Na1—O3W—Na3^v^	77.91 (2)
O4W^i^—Na1—O5W^ii^	80.67 (2)	Na2—O3W—H3WA	99.0 (11)
O1W—Na2—O6W	161.83 (3)	Na1—O3W—H3WA	123.6 (14)
O1W—Na2—O7W	103.70 (3)	Na3^v^—O3W—H3WA	91.4 (11)
O6W—Na2—O7W	90.51 (3)	Na2—O3W—H3WB	99.4 (10)
O1W—Na2—O3W	85.30 (3)	Na1—O3W—H3WB	126.9 (14)
O6W—Na2—O3W	104.12 (3)	Na3^v^—O3W—H3WB	98.2 (10)
O7W—Na2—O3W	98.07 (3)	H3WA—O3W—H3WB	109.3 (18)
O1W—Na2—O5W	83.00 (2)	Na3—O4W—Na1^iv^	146.50 (3)
O6W—Na2—O5W	84.44 (3)	Na3—O4W—Na2^iv^	78.47 (2)
O7W—Na2—O5W	95.43 (2)	Na1^iv^—O4W—Na2^iv^	73.20 (2)
O3W—Na2—O5W	163.89 (3)	Na3—O4W—H4WA	106.6 (12)
O1W—Na2—O4W^i^	82.79 (2)	Na1^iv^—O4W—H4WA	88.0 (12)
O6W—Na2—O4W^i^	82.97 (3)	Na2^iv^—O4W—H4WA	145.9 (13)
O7W—Na2—O4W^i^	173.46 (3)	Na3—O4W—H4WB	100.9 (14)
O3W—Na2—O4W^i^	83.17 (2)	Na1^iv^—O4W—H4WB	106.3 (13)
O5W—Na2—O4W^i^	84.41 (2)	Na2^iv^—O4W—H4WB	112.7 (13)
O4W—Na3—O7W	94.70 (3)	H4WA—O4W—H4WB	99.5 (17)
O4W—Na3—O2W^iii^	88.73 (3)	Na2—O5W—Na3^i^	81.11 (2)
O7W—Na3—O2W^iii^	116.04 (3)	Na2—O5W—Na1^vi^	155.28 (3)
O4W—Na3—O3W^iii^	171.10 (3)	Na3^i^—O5W—Na1^vi^	76.19 (2)
O7W—Na3—O3W^iii^	94.17 (3)	Na2—O5W—H5WA	95.7 (14)
O2W^iii^—Na3—O3W^iii^	88.02 (3)	Na3^i^—O5W—H5WA	119.9 (13)
O4W—Na3—O5W^iv^	90.25 (2)	Na1^vi^—O5W—H5WA	104.0 (14)
O7W—Na3—O5W^iv^	159.23 (3)	Na2—O5W—H5WB	102.5 (13)
O2W^iii^—Na3—O5W^iv^	84.18 (3)	Na3^i^—O5W—H5WB	135.9 (15)
O3W^iii^—Na3—O5W^iv^	81.18 (2)	Na1^vi^—O5W—H5WB	87.4 (14)
O4W—Na3—O6W^iv^	81.54 (2)	H5WA—O5W—H5WB	103.7 (18)
O7W—Na3—O6W^iv^	83.65 (3)	Na2—O6W—Na3^i^	78.90 (3)
O2W^iii^—Na3—O6W^iv^	158.82 (3)	Na2—O6W—H6WA	123.6 (13)
O3W^iii^—Na3—O6W^iv^	98.76 (3)	Na3^i^—O6W—H6WA	89.3 (13)
O5W^iv^—Na3—O6W^iv^	77.13 (2)	Na2—O6W—H6WB	129.9 (13)
O1—P1—O2	108.28 (3)	Na3^i^—O6W—H6WB	98.9 (13)
O1—P1—O3	111.12 (4)	H6WA—O6W—H6WB	106.2 (18)
O2—P1—O3	109.74 (4)	Na2—O7W—Na3	115.38 (3)
O1—P1—O4	109.68 (3)	Na2—O7W—H7WA	112.1 (13)
O2—P1—O4	109.87 (4)	Na3—O7W—H7WA	104.2 (14)
O3—P1—O4	108.15 (4)	Na2—O7W—H7WB	106.6 (12)
P1—O1—Na1	113.60 (4)	Na3—O7W—H7WB	113.3 (12)
Na2—O1W—Na1	82.53 (2)	H7WA—O7W—H7WB	105 (2)

Symmetry codes: (i) *x*, *y*−1, *z*; (ii) *x*+1/2, −*y*+1, *z*; (iii) *x*−1/2, −*y*+2, *z*; (iv) *x*, *y*+1, *z*; (v) *x*+1/2, −*y*+2, *z*; (vi) *x*−1/2, −*y*+1, *z*.

### Trisodium orthophosphate heptahydrate (Na3PO4H2O7) Hydrogen-bond geometry (Å, º)

**Table:**

*D*—H···*A*	*D*—H	H···*A*	*D*···*A*	*D*—H···*A*
O1*W*—H1*WA*···O4^vi^	0.84 (1)	2.09 (1)	2.9344 (9)	176 (2)
O1*W*—H1*WB*···O2^iv^	0.85 (1)	1.74 (1)	2.5865 (9)	172 (2)
O2*W*—H2*WA*···O6*W*^vii^	0.85 (1)	2.38 (1)	3.2042 (11)	163 (2)
O2*W*—H2*WB*···O4^iv^	0.84 (1)	1.93 (1)	2.7634 (9)	175 (2)
O3*W*—H3*WA*···O1^viii^	0.85 (1)	1.85 (1)	2.6867 (9)	168 (2)
O3*W*—H3*WB*···O3^ix^	0.87 (1)	1.88 (1)	2.7391 (9)	172 (2)
O4*W*—H4*WA*···O2^iv^	0.84 (1)	1.97 (1)	2.8058 (9)	173 (2)
O4*W*—H4*WB*···O3^ix^	0.86 (1)	1.84 (1)	2.6978 (9)	171 (2)
O5*W*—H5*WA*···O3^x^	0.85 (1)	1.87 (1)	2.7114 (9)	173 (2)
O5*W*—H5*WB*···O4^vi^	0.85 (1)	1.92 (1)	2.7544 (9)	169 (2)
O6*W*—H6*WA*···O1^x^	0.85 (1)	1.99 (1)	2.8109 (9)	163 (2)
O6*W*—H6*WB*···O4^viii^	0.84 (1)	2.01 (1)	2.8323 (9)	169 (2)
O7*W*—H7*WA*···O4^ix^	0.85 (1)	2.02 (1)	2.8616 (9)	170 (2)
O7*W*—H7*WB*···O2^x^	0.86 (1)	1.93 (1)	2.7894 (9)	175 (2)

Symmetry codes: (iv) *x*, *y*+1, *z*; (vi) *x*−1/2, −*y*+1, *z*; (vii) −*x*+3/2, *y*, *z*−1/2; (viii) −*x*+3/2, *y*, *z*+1/2; (ix) −*x*+3/2, *y*+1, *z*+1/2; (x) −*x*+1, −*y*+1, *z*+1/2.
